# Intercellular Molecular Crosstalk Networks within Invasive and Immunosuppressive Tumor Microenvironment Subtypes Associated with Clinical Outcomes in Four Cancer Types

**DOI:** 10.3390/biomedicines11113057

**Published:** 2023-11-14

**Authors:** Jinfen Wei, Wenqi Yu, Lei Wu, Zixi Chen, Guanda Huang, Meiling Hu, Hongli Du

**Affiliations:** School of Biology and Biological Engineering, South China University of Technology, Guangzhou 510006, China; weijinfen@scut.edu.cn (J.W.); 202121049993@mail.scut.edu.cn (W.Y.); wlei0928@163.com (L.W.); bizic_chen@mail.scut.edu.cn (Z.C.); 202010108432@mail.scut.edu.cn (G.H.); melody931109@sina.com (M.H.)

**Keywords:** tumor microenvironment subtypes, tumor invasion, single-cell RNA-seq, immunosuppressive, cell-cell interaction, *COL1A1-SDC1*, *C5AR1-RPS19*, *LGALS9-HAVCR2*, *SPP1-PTGER4*, *COL6A3-SDC1*

## Abstract

Heterogeneity is a critical basis for understanding how the tumor microenvironment (TME) contributes to tumor progression. However, an understanding of the specific characteristics and functions of TME subtypes (subTMEs) in the progression of cancer is required for further investigations into single-cell resolutions. Here, we analyzed single-cell RNA sequencing data of 250 clinical samples with more than 200,000 cells analyzed in each cancer datum. Based on the construction of an intercellular infiltration model and unsupervised clustering analysis, four, three, three, and four subTMEs were revealed in breast, colorectal, esophageal, and pancreatic cancer, respectively. Among the subTMEs, the immune-suppressive subTME (subTME-IS) and matrix remodeling with malignant cells subTME (subTME-MRM) were highly enriched in tumors, whereas the immune cell infiltration subTME (subTME-ICI) and precancerous state of epithelial cells subTME (subTME-PSE) were less in tumors, compared with paracancerous tissues. We detected and compared genes encoding cytokines, chemokines, cytotoxic mediators, PD1, and PD-L1. The results showed that these genes were specifically overexpressed in different cell types, and, compared with normal tissues, they were upregulated in tumor-derived cells. In addition, compared with other subTMEs, the expression levels of *PDCD1* and *TGFB1* were higher in subTME-IS. The Cox proportional risk regression model was further constructed to identify possible prognostic markers in each subTME across four cancer types. Cell-cell interaction analysis revealed the distinguishing features in molecular pairs among different subTMEs. Notably, ligand–receptor gene pairs, including *COL1A1-SDC1*, *COL6A2-SDC1*, *COL6A3-SDC1*, and *COL4A1-ITGA2* between stromal and tumor cells, associated with tumor invasion phenotypes, poor patient prognoses, and tumor advanced progression, were revealed in subTME-MRM. *C5AR1-RPS19*, *LGALS9-HAVCR2*, and *SPP1-PTGER4* between macrophages and CD8+ T cells, associated with CD8+ T-cell dysfunction, immunosuppressive status, and tumor advanced progression, were revealed in subTME-IS. The spatial co-location information of cellular and molecular interactions was further verified by spatial transcriptome data from colorectal cancer clinical samples. Overall, our study revealed the heterogeneity within the TME, highlighting the potential pro-invasion and pro-immunosuppressive functions and cellular infiltration characteristics of specific subTMEs, and also identified the key cellular and molecular interactions that might be associated with the survival, invasion, immune escape, and classification of cancer patients across four cancer types.

## 1. Introduction

The importance of the tumor microenvironment (TME) for malignant progression is well recognized across cancer types [1,2,3]. The components of the TME, including cancer-associated fibroblasts (CAFs) [4], tumor-associated macrophages (TAMs) [5], and endothelial cells [6], provide specialized microenvironments for cancer cells and influence tumor progression. Recently, studies based on bulk RNA-seq data have shown that specific TME subtypes are related to tumor metastasis [7], immune checkpoint therapy treatment response [1], malignant progression [8], and poor prognosis [9]. However, the specific cell types and their molecular characteristics involved in TME subtypes need to be clearly revealed. 

Single-cell RNA sequencing (scRNA) technology has made it possible to analyze the heterogeneity of a TME, where the cells in the TME directly influence the biological characteristics of other cells through paracrine pathways, further affecting tumor progression. Recent studies revealed remarkable cellular complexities in TME, comprising numerous stromal cell subpopulations, especially in the CAF [10], TAM [11], and T-cell [12] compartments. We are also learning that CAFs and TAMs display unique functions in promoting or restraining tumors. COX2^+^ CAFs are involved in extracellular matrix (ECM) remodeling and promoting lung cancer metastasis [13]. ATF4^+^ CAFs promote tumor angiogenesis and metastasis by regulating the expression of collagen genes in pancreatic ductal adenocarcinoma (PDAC) [14]. LRRC15^+^ CAFs suppress tumor immunity, reduces the response to immunotherapy, and drives tumor growth [15]. STAB1^+^TREM2^+^ TAMs were revealed to have immune-suppressive capacities in patients resistant to immune checkpoint blockade [16]. Extracellular vesicles released by tumor cells specifically promote pro-inflammatory macrophages, further facilitate T-cell infiltration, and extend patient survival [17]. Research based on different subclusters shows that CD169^+^ TAMs in human and mouse gliomas produce pro-inflammatory chemokines, leading to the accumulation of T cells and NK cells and further suppressing tumor growth [18]. TREM2^+^ TAMs, most enriched in patients and corresponding to disease grade, restrain anti-tumor immunity by affecting the infiltration and effector functions of CD8+ T cells in ovarian cancer [19]. 

Our previous research has shown that SPP1^+^ TAMs and APOE^+^CYSZ^+^ TAMs are involved in regulating tumor invasion phenotypes and glycolysis in lung cancer [20] and the recruitment of T regulatory (Treg) cells and formation of an immunosuppressive TME in colorectal cancer (CRC) [21], respectively. However, due to the heterogeneity of each cell type in TMEs, multiple cells show diverse functions; it is difficult to define whether the function of a microenvironment is pro-tumor or anti-tumor. In addition, the interactions between tumor and stroma cells, as well as immune cells, are not uniformly tumor-promoting or inhibitory, and the underlying intricate differences remain to be fully revealed. 

Given that the cell infiltration level can reflect the composition of a microenvironment to establish its function [12], we hypothesized that cell clusters with co-infiltration are distinguishable subtypes within the TME and wanted to identify and define the function of TME subtypes in tumors. Based on scRNA-seq data of samples (more than 20 samples with clear clinical information in each dataset) among breast cancer (BC), CRC, esophageal cancer (ESCA), and PDAC from previously published data, our study systematically analyzed cellular co-infiltration patterns to distinguish TME subtypes and defined their pro- or anti-tumor functions in each TME subtype. We then extensively predicted cell-cell interaction (CCI) signals between cell-type pairs in each TME subtype to reveal specific CCI signals associated with tumor progression. By analyzing the pathways involved in CCI signals, we identified the key signaling pathways that influence malignant phenotypes and TME subtype functions to further explore the potential therapeutic targets. In addition, we also revealed similarities and heterogeneities of TME subtypes and CCI pairs among the tumor types.

## 2. Materials and Methods

### 2.1. Data Collection

The single-cell datasets used in the present study were downloaded from the previous studies in the following databases by accession number: CRC [22] (Single Cell Portal, SCP1162), BC [23] (Gene expression Omnibus, GSE123814), PDAC [24] (Genome Sequence Archive, PRJCA001063), ESCA [25] (Gene expression Omnibus, GSE160269), RCC [26] (Sequence Read Archive, SRZ190804), and LUAD [27] (Gene expression Omnibus, GSE131907). Bulk transcriptome datasets of CRC, BRCA, PDAC, and ESCA were downloaded from The Cancer Genome Atlas (TCGA). 

### 2.2. Single-Cell RNA-Seq Data Processing and Cell Type Annotation

The raw gene expression matrices were processed using R package Seurat (v.4.2.0). In the quality control steps, the following genes or cells were eliminated: (1) genes expressed by <50 cells; (2) cells < 200 or cells > 6000 expressed genes; (3) cells > 20% of mitochondrial genes. After filtering, 189,176 and 124,380, 203,084 and 75,042, 185,814 and 16,265, and 34,842 and 14,019 high-quality cells from tumor and normal tissues in BC, CRC, ESCA, and PDAC, respectively, were preserved for subsequent analysis.

The NormalizeData function was used to divide the unique molecular identifier (UMI) counts of each gene by the total UMI count of each cell. The FindVariableFeatures function with default parameters was used to find highly variable genes. The top 2000 variably expressed genes were then used to construct principal components (PCs) using the RunPCA function with a parameter feature set. The first 30 PCs were identified and analyzed for shared nearest-neighbor graphs and unsupervised clustering with the FindNeighbors and FindClusters functions. The resolution for each cluster and subcluster analysis is presented in Supplementary Table S1. The RunUMAP function was used for nonlinear dimensionality reduction and two-dimensional visualization of the clusters. 

The FindAllMarkers function was used to analyze and identify the differentially expressed genes (DEGs) of each cluster using the default nonparametric Wilcoxon rank sum test with Bonferroni correction. DEG lists were filtered based on the following criteria: expressed in at least 50% of cluster cells; expression fold change > 1 and FDR q value < 0.05. The clusters and subclusters were annotated based on the top ranking among the DEGs known from previous studies. Detailed information on clusters, including names, cell numbers, and cell proportions, is presented in Supplementary Table S2.

### 2.3. Identification of subTMEs

To examine the potential cellular compositions of different TME ecosystems in pan-cancer, we investigated the coexistence patterns of different cell subclusters. For the cell subtype abundance correlation matrix, we defined the number ratio of cell subtype to the belonging major cell type as the relative abundance of each cell subtype, as a previous study showed [28]. Pairwise correlation values between the normalized abundance of any two cell subtypes across different tumor samples were calculated using the Spearman correlation method as shown in previous studies [29,30]. These values were then clustered using the R package tidyverse (v.1.3.2). 

### 2.4. Calculation of the Score of subTMEs 

To evaluate the level of each subTME in samples, the subTME score was defined and calculated. First, signature genes for each cell subtype were defined according to the following formula:

$$S_{g_{i}}=1-\frac{Q_{g_{i}}}{P_{g_{i}}}$$

where $S_{g_{i}}$ is the score of gene $g_{i}$ in ${sub}_{j}$ cell subtype, $P_{g_{i}}$ is the cell proportion of cells with expression of gene $g_{i}$ in cell subtype ${sub}_{j}$, and $Q_{g_{i}}$ is the cell proportion of cells with expression of gene $g_{i}$ in other cell types except for ${sub}_{j}$. 

Here, highly expressed gene cells were defined as cells whose expression of the gene was greater than the 1/4 quantile of the expression values in this cell subtype (ignoring zero values). Genes with an $S_{g_{i}}$ score greater than 0.7 were selected as the characteristic gene set for the current cell subtype. 

Second, each major cell type in the TME contains j cell subtypes, with *n* signature genes in each subtype. The cell subtype score was defined according to the following formula:

$$S_{{sub}_{j}}=\frac{\sum_{i=1}^{n}S_{g_{i}}}{n}$$

where $S_{{sub}_{j}}$ is the score of cell subtype ${sub}_{j}$, $S_{g_{i}}$ is the score of each signature gene $g_{i}$ in the current cell subtype, and *n* is the number of genes in each signature.

Finally, the subTME score was defined according to the following formula:

$$S_{subTME}=\frac{\sum_{j=1}^{n}\sqrt{S_{{sub}_{j}}\times P_{{sub}_{j}}}}{n}$$

where $S_{subTME}$ is the score for each subTME, each subTME contains *j* cell types, $P_{{sub}_{j}}$ is the cell proportion of each subtype in the major cell type to which it belongs, and *n* is the number of cell subtypes in each signature. 

### 2.5. Classification of subTMEs for Bulk RNA-Seq Data

To apply our single-cell-based subTMEs to bulk RNA-seq data, we defined gene signatures for each subtype by combining the top 10 DEGs of all clusters in the corresponding cellular subTME. For each patient, each cell type signature of a subTME was computed, and the mean value of the score was calculated across all cell type signatures within one subTME. 

### 2.6. Construction of the Cox Proportional Risk Regression Model and Prognostic Analysis

Univariate Cox regression analysis was conducted on marker genes of subTMEs screened from scRNA-seq data using the survival R package (v.3.5_7) to identify genes associated with prognosis (*p* < 0.05). The gene expression levels and corresponding regression coefficients were used to calculate the risk score of each sample. The risk formula is as follows: Risk score = ΣExp(mRNAx) × coefx, with Exp(mRNAx) and coefx representing the expression of the respective gene x and the corresponding risk coefficient. Then, patients were divided into high-score groups and low-score groups according to the median risk score. Receiver operating characteristic curve (ROC) analysis was performed using the TCGA patients to evaluate the specificity and sensitivity of the risk score in the prognostic model.

### 2.7. Cell-Cell Interaction Analysis

To investigate CCIs among cell subtypes in each subTME, we analyzed the L-R pairs using CellphoneDB (v.4.1.3) [31] and CellChat (v.1.5.0) [32] and their accompanying curated interaction database. For input and analysis data, we used the default parameter. Only significant interactions (*p*-value < 0.05) were used for further analysis. For each L-R pair, the total incidence of the L-R pair across cell subtypes from the same subTME was counted. To ensure high-confidence interactions, we subsampled cells for each cell subtype and calculated interaction scores for all L-R pairs over all cell subtype pairs as described previously [33]. In order to extract the most relevant pairs, the 100 highest-scoring L-R interactions for each cell subtype were extracted, followed by the identification of the L-R pairs with the highest coefficient of variation between all cell subtype pairs. Of these, L-R pairs were manually selected for plotting based on the solidity of the literature evidence and the biological interpretability of the interaction.

### 2.8. Gene Signature Score

Multiple gene signature scores were calculated on the basis of the scRNA-seq data. For each gene signature as previously defined, an individual cell was scored using the single sample gene set enrichment analysis (ssGSEA) method in the GSVA package (v.1.38.2). The detailed gene signatures are addressed in Supplementary Table S3.

### 2.9. Spatial Transcriptome Data Analysis

The CRC ST data sequences used in the current study were obtained in a previous study using the Stereo-seq platform [34]. The analysis process referred to our previous research [21]. For determining the co-localization of ecm_myCAF and Cancer_Malig as well as Macro_APOE and CD8Teff_2, the signature score was calculated by ssGSEA across all spots in two CRC tissue sections. The signature score of these cell types was calculated using Spearman correlation. 

### 2.10. Statistical Analysis 

All statistical analyses and graphical representations of data were performed in the R (v.4.1.3) and Python (v.3.7) computational environment. The correlation analyses including gene expression, gene signature score, and cell proportion between two groups used in this study were based on Spearman correlation. Wilcoxon tests were used to compare measurements between two groups. Kruskal-Wallis tests were used for the comparison among three or more than three groups. Adjusted *p* values < 0.05 were considered statistically significant.

## 3. Results

### 3.1. Cellular Co-Infiltration Pattern Identifies TME Subtypes across Four Cancer Types

**Figure 1:**
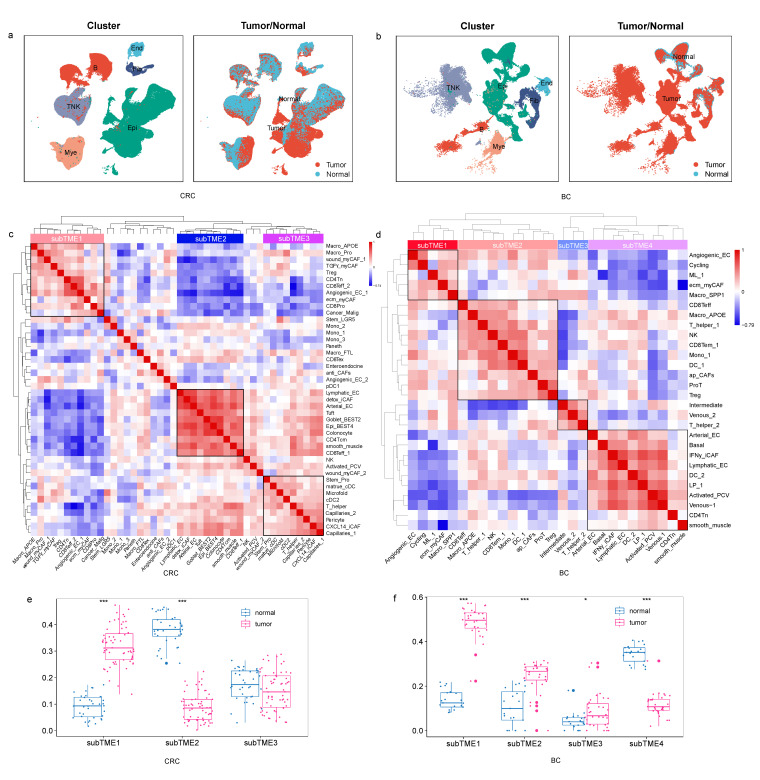
TME subtypes across cancers. (**a**) UMAP plots of cells from normal and tumor tissue of CRC patients, showing 6 clusters indicating major cell types and 2 clusters indicating cells derived from tumor and normal tissues. Each cluster is shown in a different color. (**b**) The same as shown in a but in BC patients. (**c**) The three cellular modules on the basis of correlations of cell subclusters from tumors with positive (Spearman correlation; correlation coefficient r > 0.3 and FDR < 0.05, in red), negative (r < −0.3 and FDR < 0.05, in blue), or non-significant (white) pairwise correlation for infiltration in CRC samples. (**d**) The four cellular modules on the basis of correlations of cell subclusters from tumors in BC samples. (**e**,**f**) subTME score in normal and tumor sample groups in CRC and BC. *** *p* < 0.001, * *p* < 0.05, Wilcoxon tests.

To survey the TME landscape covering all cell populations across pan-cancer, we performed scRNA-seq analysis of a total of 178 tumor and 72 normal samples, including 32 tumor and 21 normal samples in BC, 62 tumor and 36 normal samples in CRC, 60 tumor and 4 normal samples in ESCA, and 24 tumor and 11 normal samples in PDAC. After clustering and annotation, 6 major cell types (Figure 1a,b, Supplementary Figure S1a,b) and 39, 44, 61, and 72 cell subtypes (Supplementary Table S2) were identified among 313,556, 278,126, 202,079, and 48,861 cells in BC, CRC, ESCA, and PDAC, respectively. Myeloid cells were within the range of 10~17%, and epithelial cells were within the range of 21~68%. Notably, there is a significant difference in T-lymphocyte infiltration levels across cancer types. The average cell proportion of CD8+T and CD4+T was 5.6% and 7.8% in BC, 14% and 11% in CRC, 17% and 16% in ESCA, and 2.4 and 4.7% in PDAC (Supplementary Table S2). In addition, there is also significant heterogeneity in T-lymphocyte infiltration levels across samples in the same cancer type.

To investigate the characteristics of pan-cancer subTMEs, we examined co-infiltration patterns of cells from all samples in each cancer type. After calculating the proportion of cell subtypes (Supplementary Table S4), the correlation coefficient between cell proportions was calculated using Spearman correlation. Hierarchy clustering identified four, three, three, and four stable cellular modules in BC, CRC, ESCA, and PDAC, respectively (Figure 1c,d, Supplementary Figure S1c,d). On the basis of differential enrichment between subTMEs, we calculated the subTME scores across samples. As the subTME score was higher or lower in cancer compared with normal tissues, we found that a specific subTME was tumor-enriched or normal-enriched, respectively (Figure 1e,f, Supplementary Figure S1e,f). We then calculated the subTME scores in bulk RNA-seq data and found the score pattern of subTMEs in bulk RNA-seq data was consistent with the results in single-cell data (Supplementary Figure S2a). 

In order to verify the robustness of the method in identifying a subTME, we performed the same analysis with the scRNA-seq datasets of renal cell carcinoma (RCC) and lung cancer (LC) and found that the specific subTME was also enriched in cancer and normal tissues, both in single-cell and bulk data (Supplementary Figure S2b–e).

### 3.2. Different Molecular Characteristics between TME Subtypes

**Figure 2:**
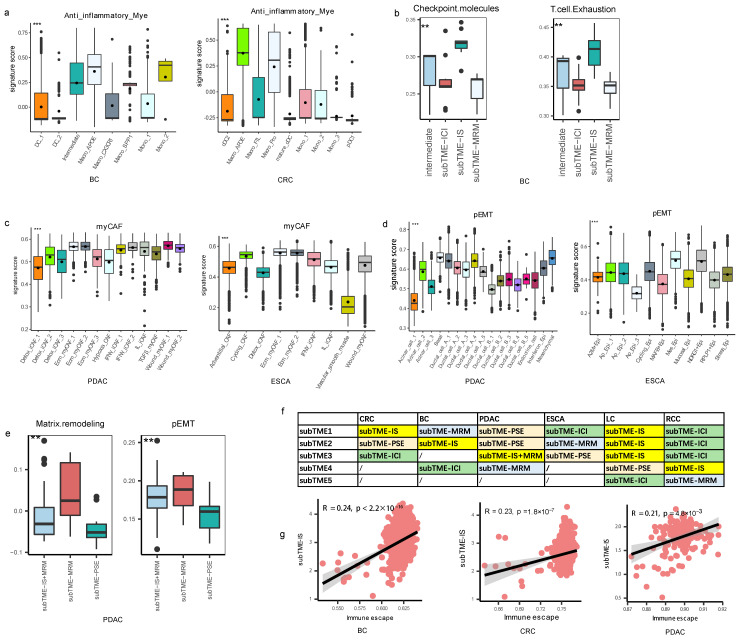
Definition of TME subtypes. (**a**) Boxplot showing the anti-inflammatory score among myeloid cells in BC and CRC samples. *** *p* < 0.001, Kruskal–Wallis test. (**b**) Boxplot showing the checkpoint molecules and T-cell exhaustion score among subTMEs in BC. ** *p* < 0.01, Kruskal–Wallis test. (**c**) Boxplot showing the myCAF score among fibroblasts in PDAC and ESCA samples. *** *p* < 0.001, Kruskal–Wallis test. (**d**) Boxplot showing the pEMT score among epithelial cells in PDAC and ESCA samples. *** *p* < 0.001, Kruskal–Wallis test. (**e**) Boxplot showing the matrix remodeling and EMT score among subTMEs in PDAC. ** *p* < 0.01, Kruskal–Wallis test. (**f**) Definitions of the TME subtypes across cancers. (**g**) Scatterplot showing the Spearman correlation between immune escape score and subTME-IS score in BC, CRC, and PDAC, the error band indicates 95% confidence interval.

The subTME including subTME1 in CRC and subTME2 in BC was found to have the enrichment of Macro_APOE, Treg, and CD8+ Tex cells, and TAM subtypes in this subTME, such as Macro_APOE, harbored high anti-inflammatory signatures (Figure 2a). Among them, Treg cells are a well-known type of immunosuppressive cells. In addition, the high score of signatures “Checkpoint molecules” and “T cell Exhaustion” were higher in this subTME (Figure 2b). These results suggested that the current subTME exhibited an immunosuppression state, and it was designated as subTME-IS (immune-suppressive) (Figure 2f). 

The subTME including subTME1 in BC, subTME2 in ESCA, subTME4 in PDAC, and subTME5 in RCC was enriched by stromal cells including Angiogenic_EC, ecm_myCAF, Macro_SPP1, and malignant epithelial cells. CAFs in this subTME harbored a myCAF signature (Figure 2c), and epithelial cells harbored a partial epithelial-to-mesenchymal transition (pEMT) signature (Figure 2d). For example, the pEMT score was highest in NDRG1+ Epi of ESCA and in Ductal_cell_A-type cells of PDAC among epithelial cells. Enriched signatures of “Matrix remodeling” and “epithelial-to-mesenchymal transition (EMT)” in this subTME (Figure 2e) led us to designate this subTME as subTME-MRM (matrix remodeling with malignant cells) (Figure 2f). 

The subTME including subTME3 in CRC, subTME4 in BC, subTME1 in ESCA, and subTME1/2/3 in RCC contained activated myeloid and endothelial cells, including mature dendritic cells (mature DCs), conventional DCs (cDCs), NK cells, T helpers, and monocytes, along with capillaries, suggesting that this dominant TME exhibited a normal-order angiogenesis process and an immune cell infiltration state. The high expression of the signature “Myeloid cells traffic” was higher in this subTME (Supplementary Figure S3a); therefore, this subTME was designated as subTME-ICI (immune cell infiltration) (Figure 2f). 

The subTME including subTME2 in CRC, subTME3 in ESCA, and subTME1 in PDAC mainly contained non-malignant epithelial cells but fewer infiltrating immune cells, and the signature of “Epithelial cell” (Supplementary Figure S3b) was higher in this subTME, which was designated as subTME-PSE (precancerous state of epithelial cells) (Figure 2f). 

We named this classification for “TME subtypes at the single-cell resolution including immune suppressive, matrix remodeling with malignant cells, immune cell infiltration and precancerous state of epithelial cells”. Further, we calculated the correlation between subTME scores and tumor-related gene signature scores in bulk RNA-seq datasets corresponding to cancer types in single cells. The results showed that the “immune escape signature” score was correlated with the subTME-IS score in CRC, BC, and PDAC (Figure 2g). Matrix remodeling, tumor proliferation, angiogenesis, and EMT score were significantly positively correlated with the subTME-MRM score in BC, ESCA, and PDAC (Supplementary Figure S3c–e). These results further validated our definition of subTMEs and showed that subTME-derived signatures could also be used for bulk data. Based on the above results, there were two tumor-specific subTMEs (subTME-IS, subTME-MRM) and two paracancerous-specific subTMEs (subTME-PSE, subTME-ICI). 

As cytokines, chemokines, cytotoxic mediators, and immune checkpoints are the ultimate factors in suppressing or promoting a tumor, we analyzed the differences in these factors (Supplementary Table S5) between various TME subtypes. Firstly, we detected the gene expression of genes encoding cytokines, chemokines, cytotoxic mediators, and immune checkpoints in each cell type (Supplementary Table S5, Supplementary Figures S4 and S5). As revealed by prior knowledge, cytotoxic mediators were mainly expressed in T lymphocytes and natural killer cells; cytokines, including *IL10*, *IL1A*, *IL1B*, and *IL18*, and chemokines, including *CXCL8*, *CXCL9*, *CXCL10,* and *CCL18*, were mainly expressed in myeloid cells; *CXCL17* was mainly expressed in epithelial cells; *CSF3*, *TNFSF10*, and *CX3CL1* were mainly expressed in endothelial cells; *CXCL14* and *CXCL12* were mainly expressed in fibroblasts; other genes were widely expressed in various cell types.

Secondly, we analyzed the differential expression of genes in specific cells mentioned above between cancer and normal tissues. Compared with normal tissues, *PDCD1*, *CD247*, *CXCL2*, *CXCL3*, *CXCL16*, *CXCL17*, *CCL20*, and *TGFB1* were highly expressed in tumor epithelial cells (Supplementary Figure S5); *PDCD1*, *CD247*, *CCL4*, *CCL5, GZMB*, *GZMA*, *IFNG*, *NKG7*, *XCL1*, and *XCL2* were highly expressed in tumor T lymphocytes and natural killer cells (Supplementary Figure S6); *PDCD1*, *CD247*, *CCL18*, *TNF*, *CSF1*, *IL10*, and *IL1A* were highly expressed with tumor myeloid cells (Supplementary Figure S7); *TGFB1* was highly in tumor fibroblasts (Supplementary Figure S8); and *CSF3* was highly expressed in endothelial cells (Supplementary Figure S9). Thirdly, we analyzed and compared the expression levels of these genes between different TME subtypes. *PDCD1*, *TGFB1*, *CXCL8*, *CCL3*, *CCL4*, and *CCL5* were highly expressed in subTME-IS; *CXCL2* and *CXCL12* were highly expressed in subTME-ICI, which was consistent among cancer types (Supplementary Figure S10). However, other factors exhibited heterogeneity between the four cancer types.

These results indicate that compared with normal tissues, these cytokines, chemokines, and cytotoxic mediators exhibit abnormal expression in various cell types derived from cancer tissues. However, there is a need for more multilevel data to study the relationship between these factors within the TME subtypes, to further determine whether anti-tumor or pro-tumor effects are activated or suppressed in the current TME. 

### 3.3. Construction of TME-Subtype-Based Risk Model 

**Figure 3:**
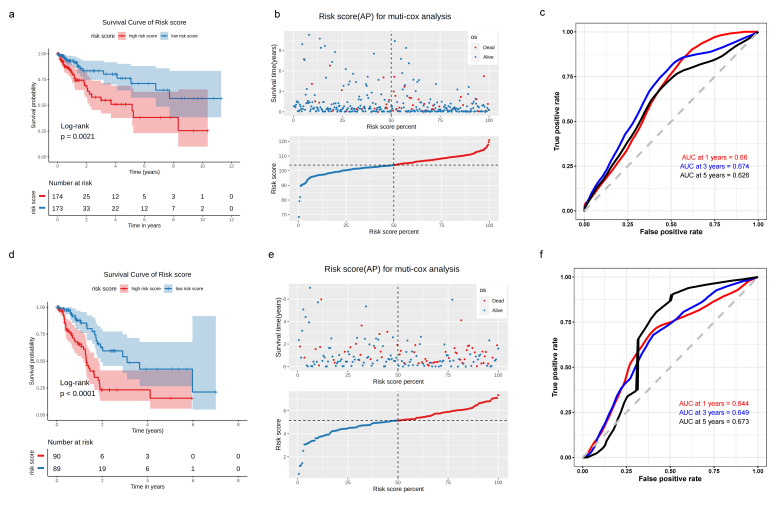
KM survival analysis, risk score assessment using the subTME gene markers, and time-dependent ROC curves in CRC and PDAC datasets. (**a**) KM survival analysis of high- and low-risk samples. (**b**) Relationship between the survival status/risk score rank and survival time/risk score rank. (**c**) Time-dependent ROC curve for overall survival of the CRC datasets. (**d**–**f**) The same as in (**a**–**c**) but in PDAC datasets.

As the subTME score was correlated with the clinicopathological characteristics, we used subTME-related marker genes to further evaluate the impact of subTMEs on the prognosis of tumor patients in each cancer type. Among the markers in subTME1, subTME1, subTME2, and subTME4 in BC, CRC, ESCA, and PDAC, respectively, the survival-related genes were selected to construct the risk model (*p* < 0.05, Supplementary Table S6). The risk score of CRC subTME1 was calculated as follows: risk score = (−0.666 × DIAPH2) + (−0.654 × TNFRSF9) + (0.735 × SH3BGRL3) + (0.138 × RPS4Y1) + (0.771 × SLC11A2) + (1.210 × RPS4X) + (0.987 × NDUFA11) + (0.565 × SLC43A3) + (−1.506 × RER1) + (−1.576 × ACTG1) + (0.991 × FAM3C). Kaplan–Meier (KM) survival analysis based on this regression model indicated that the prognosis of CRC patients in the low-risk group was better than that in the high-risk group (*p* = 0.0021, Figure 3a,b). Furthermore, ROC curves were plotted to assess the sensitivity and specificity of the risk model. The area under the curve (AUC) values of the 1-year, 3-year, and 5-year ROC curves were 0.66, 0.674, and 0.626, respectively, in CRC samples (Figure 3c). As shown in Figure 3d, the prognosis of the low-risk group was better than that of the high-risk group in PDAC patients (*p* < 0.0001), and the AUC values of the 1-year, 3-year, and 5-year ROC curves were 0.644, 0.649, and 0.673, respectively, in PDAC samples (Figure 3f). Correspondingly, the subTMEs of BC and ESCA were constructed based on marker genes of each subTME (Supplementary Figure S11a–f). 

### 3.4. TME Subtypes Exhibit Distinct Features in Cell-Cell Interaction Pairs

**Figure 4:**
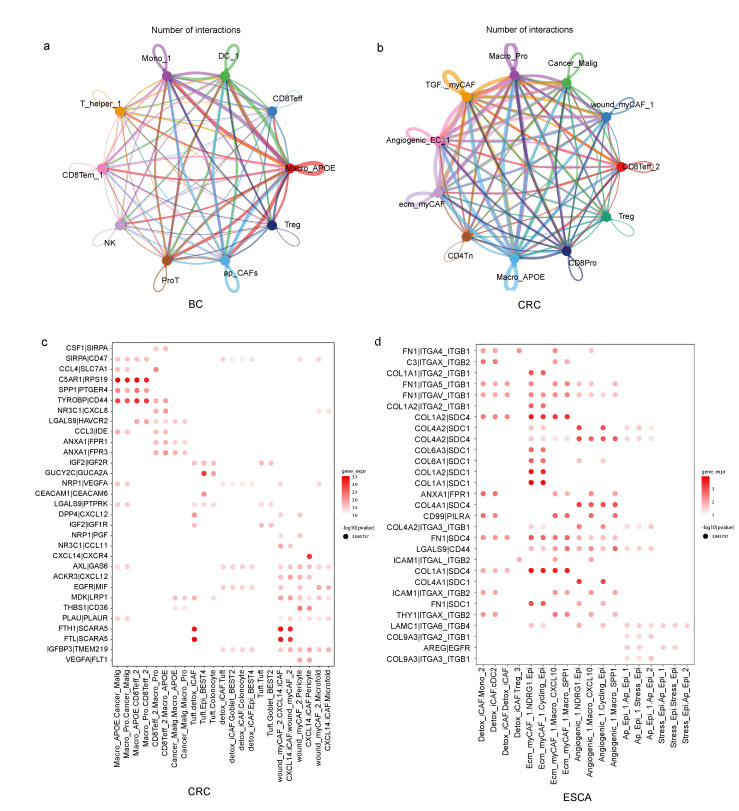
Cell-cell interaction across cell subclusters within TME subtypes. (**a**) Social graph depicting the number of interactions between cell types within subTME-IS in BC. (**b**) Social graph depicting the number of interactions between cell types within subTME-IS in CRC. (**c**) Enrichment of selected ligand-receptor interactions in CRC for the cell-type pairs within subTME-IS. (**d**) Enrichment of selected ligand–receptor interactions in ESCA for the cell-type pairs within subTME-MRM. The bubbles shown in the figure indicate *p* < 0.05; the color of the bubbles indicates the level of mean gene expression of ligand–receptor.

To examine how specific cells interacted with other cells and potentially affected the biological characteristics of the belonging subTME in cancer development, we conducted a CCI analysis within subTMEs using the cellphoneDB and CellChat method in four cancer types. We first quantified the total number of predicted ligand-receptor (L-R) interactions for all cell-type pairs in each subTME, and we found that there were differences in the number of interactions among cells within the same subTME. For example, TAMs (Macro_APOE in BC and CRC) were one of the cell types that communicated more frequently with other cell types in subTME-IS (Figure 4a,b). Angiogenicss_EC, ecm_myCAF, and Macro_SPP1 were the cell types that communicated more frequently with other cell types in subTME-MRM across BC, PDAC, and ESCA (Supplementary Figure S12a–c). Particularly, ecm_myCAF had more interactions with malignant tumor cells than other cell types. 

To compare the difference in molecular characteristics in predicted interactions among subTMEs, we conducted a gene enrichment analysis using L-R pairs in each subTME. The results showed that the specific genes in L-R pairs were significantly enriched in interleukin-10 signaling, cell surface interactions at the vascular wall, cytokine signaling in the immune system, and signaling by receptor tyrosine kinases in subTME-IS, subTME-MRM, subTME-ICI, and subTME-PSE, respectively (Supplementary Figure S12d–g). These results suggested the cell composition and CCI signal between cells together created the biological characteristics of each subTME.

To further separate cell-subtype-specific from ubiquitous interactions, we used the coefficient of variation of the 100 top-scoring predicted L-R interactions for each of the main cell-type pairs and thus identified pair-specific interactions in each subTME. Then, pairs were manually selected for plotting based on the reliability of the literature research on the biological significance of the interaction in cancer (Figure 4c,d, Supplementary Figure S13a–f).

### 3.5. Invasive States of Tumor Cells Related to Cell-Cell Interaction Patterns in subTME-MRM

We systematically investigated how cellular interaction differed in tumor-enriched subTMEs (subTME-MRM and subTME-IS), and the specific function of intercellular communication genes was further analyzed in depth. 

**Figure 5:**
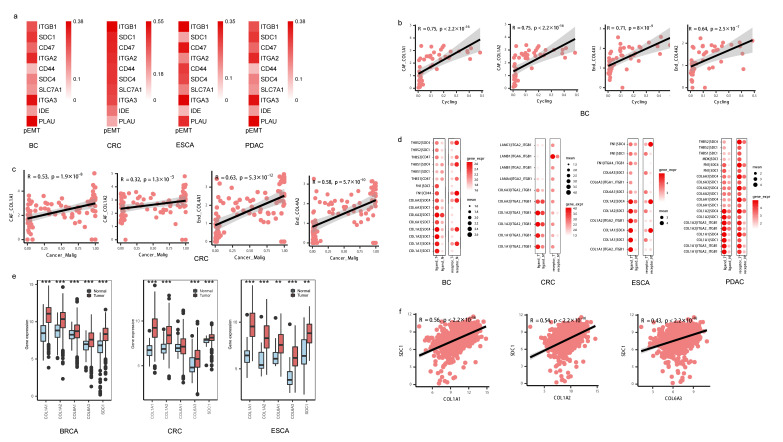
EMT phenotype of tumor cells regulated by stromal cells. (**a**) Heatmap showing the Spearman correlation between gene expression and pEMT score in tumor cells, *p* < 0.05. (**b**,**c**) Scatterplot showing the Spearman correlation of the gene expression in fibroblasts and cell proportion of tumor cells in BC and CRC, the error band indicates 95% confidence interval. (**d**) Bubble chart showing the gene expression of ligand–receptor pairs between normal and tumor samples derived from fibroblasts and tumor cells, respectively, in single-cell data; the color of the bubbles indicates the level of gene expression of a ligand or receptor in sender or receiver cells; the bubbles shown in the figure indicate *p* < 0.05, Wilcoxon tests. (**e**) Boxplot showing the gene expression of ligand–receptor pairs between normal and tumor samples in TCGA data, *** *p* < 0.001, ** *p* < 0.01, Wilcoxon tests. (**f**) Scatterplot showing the Spearman correlation of the gene expression in TCGA-BC tumor data.

As myCAF and Angiogenic_EC frequently interacted with malignant cells, we focused on these cell subtypes to investigate the potential biological effects on the phenotype of malignant cells. We found that the key genes, especially *SDC1*, *SDC4*, *ITGA2*, and *ITGA3*, in malignant cells that interacted with CAFs and Angiogenic_EC were positively correlated with the pEMT score in tumor cells (Figure 5a). 

To investigate whether the pEMT biological process is potentially caused by SDC1, SDC4, ITGA2, and ITGA3 expressed by CAFs, we further focused on the correlation between the ligand genes from CAFs as well as from Angiogenic_EC with the abundance of malignant tumor cells undergoing pEMT. Gene expression of *COL1A1*, *COL1A2*, *COL6A3*, *FN1*, and *THY1* in CAFs as well as *COL4A1* and *COL4A2* in Angiogenic_EC had a highly significantly positive correlation with the proportion of malignant tumor cells. For example, gene expression levels of *COL1A1*, *COL6A3*, and *FN1* in CAFs as well as *COL4A1* and *COL4A2* in Angiogenic_EC were correlated with the proportion of the Cancer_Malig subtype in BC and CRC, indicating these pEMT tumor cells might be regulated by *COL1A1* in CAFs and *COL4A1* in Angiogenic_EC (Figure 5b,c, Supplementary Figure S14a, Supplementary Table S7). 

**Figure 6:**
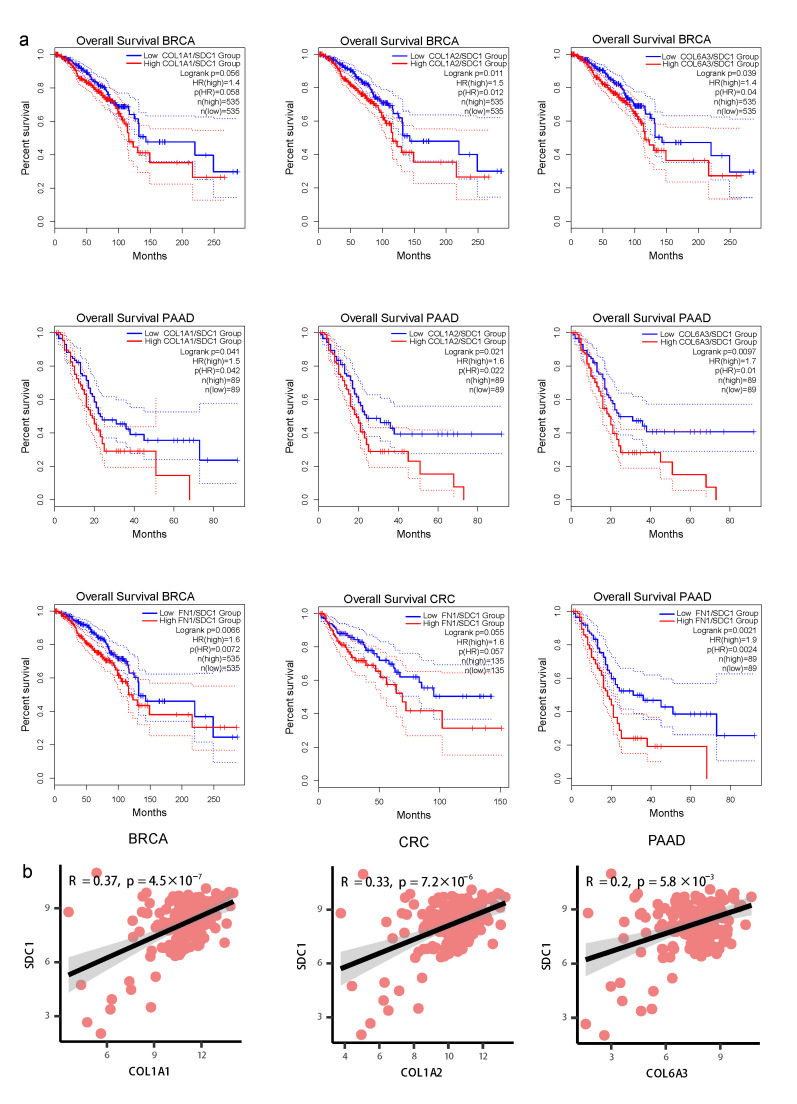
Clinical prognosis of ligand–receptor interactions between fibroblasts and tumor cells. (**a**) The Kaplan–Meier curve shows overall survival of *COL1A1*-*SDC1*, *COL1A2*-*SDC1*, and *COL6A3*-*SDC1* in BC and PDAC patients. (**b**) Scatterplot showing the Spearman correlation of the gene expression in TCGA-PDAC tumor data, the error band indicates 95% confidence interval.

We analyzed the differences in gene expression between normal and tumor samples both in single-cell and bulk data. The expression of L-R gene pairs including *COL1A2-SDC4*, *COL1A2-SDC1*, *COL6A1-SDC1*, *COL6A2-SDC1*, and *COL1A1-ITGA2_ITGB1* was higher in sender and receiver cells derived from tumor tissue compared with those from normal tissue (Figure 5d). *COL1A2*, *COL1A1*, *COL6A3*, and *SDC1* were expressed at higher levels in tumor tissue compared with normal tissue in the TCGA-BC, TCGA-CRC, and TCGA-ESCA datasets (Figure 5e). Further, *COL1A1*, *COL1A2*, *COL6A1*, *COL6A3*, and *SDC1* were upregulated in late-stage compared with early-stage patients in four cancer types (Supplementary Figure S14b). High expression levels of the *COL1A1*-*SDC1*, *COL1A2*-*SDC1*, and *COL6A3*-*SDC1* gene pairs were positively correlated with poor prognosis in PDAC and BC (Figure 6a). Furthermore, the gene expression levels of *COL1A1* with *SDC1*, *COL1A2* with *SDC1*, and *COL6A3* with *SDC1* were significantly positively correlated in TCGA-BC (Figure 5f) and TCGA-PDAC (Figure 6b), supporting their potential interaction in the surrounding tumor niche. 

Overall, these data together showed that *COL1A1*, *COL1A2*, and *COL6A3* are the key molecules that might regulate the tumor pEMT phonotype by interacting with *SDC1* and accelerate the malignant progression of cancer patients.

### 3.6. Immunosuppression States and Immune Dysfunction Caused by TAMs Related to Cell-Cell Interaction Patterns in subTME-IS

**Figure 7:**
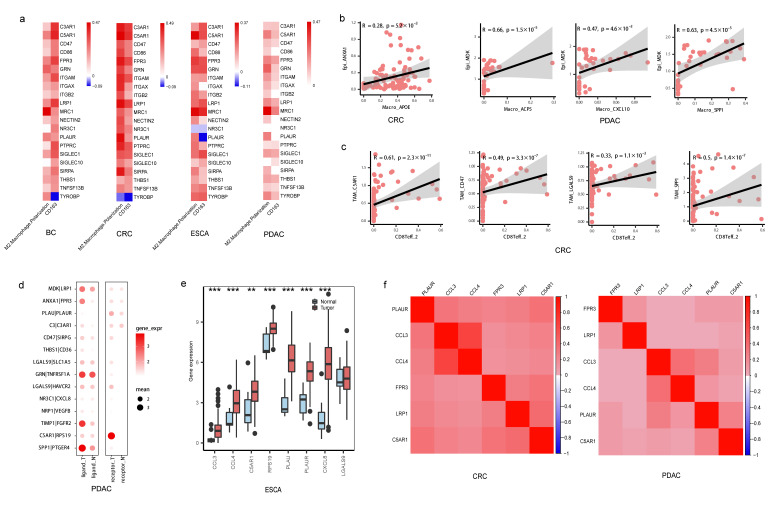
Immunosuppressive microenvironment regulated by TAMs. (**a**) Heatmap showing the Spearman correlation between gene expression and M2-like macrophage polarization score as well as CD163 expression in macrophages; color in red indicating positive (Spearman correlation; FDR < 0.05), color in blue indicating negative (FDR < 0.05), color in white indicating non-significant (FDR > 0.05). (**b**) Scatterplot showing the Spearman correlation of the gene expression in tumor cells and cell proportion of TAMs in CRC and PDAC, the error band indicates 95% confidence interval (**c**) Scatterplot showing the Spearman correlation of the gene expression in TAMs and cell proportion of CD8+ T cells in CRC, the error band indicates 95% confidence interval. (**d**) Bubble chart showing the gene expression of ligand–receptor pairs derived from tumor cells and TAMs or TAMs and CD8+ T cells, respectively, between normal and tumor samples in single-cell data in PDAC; the color of the bubbles indicates the level of gene expression of a ligand or receptor in sender or receiver cells; the bubbles shown in the figure indicate *p* < 0.05, Wilcoxon tests. (**e**) Boxplot showing the gene expression of ligand–receptor pairs between normal and tumor samples in TCGA-ESCA data, *** *p* < 0.001, ** *p* < 0.01, Wilcoxon tests. (**f**) Heatmap showing the Spearman correlation between gene expression levels in TAMs in CRC and PDAC, *p* < 0.05.

Further, we focused on the L-R interactions between tumor cells and TAMs as well as TAMs and CD8+ T cells in subTME-IS. At first, tumor cells were shown to interact with TAM subtypes, such as the *ANXA1-FPR3*, *PLAU-PLAUR*, and *MDK-LRP1* pairs between the Ductal_cell_A and Macro_SPP1_APOE cell types in PDAC. Conversely, TAMs were also shown to interact with tumor cells, suggesting there were positive-feedback loops between TAMs and tumor cells. For example, *CCL3-IDE*, *CCL4-SLC7A1*, and *C5AR1-RPS19* were predicted between Macro_APOE and Cancer_Malig in CRC. Among them, gene expression levels of *FPR3*, *PLAUR*, and *LRP1* were associated with the M2-polarization score in TAMs (Figure 7a). Genes including *ANXA1* and *PLAU* in tumor cells were positively correlated with the cell proportion of Macro_APOE in CRC, and *MDK* expression was positively correlated with the cell proportion of Macro_ACP5 in PDAC (Figure 7b, Supplementary Table S7), suggesting tumor cells might contribute to macrophage polarization within the surrounding TME. 

TAM subtypes were predicted to interact with CD8+ T cells via *SPP1-PTGER4*, *LGALS9-HAVCR2*, *CXCL8-NR3C1*, and *C5AR1-RPS19*. The gene expression levels of *CD47*, *C5AR1*, *LGALS9*, and *SPP1* were positively correlated with the cell proportion of CD8Teff_2 in CRC (Figure 7c, Supplementary Table S7). Among them, *C5AR1* expression was associated with the M2-polarization score in TAMs (Figure 7a). Notably, CD8Teff_2 harbored a high exhaustion score among T cells in CRC, suggesting this cell subtype may be the pre-exhaustion CD8+T subpopulation (Supplementary Figure S15a).

We analyzed the differences in gene expression between normal and tumor samples both from single-cell and bulk data. Gene expression levels of *ANXA1-FPR3*, *NECTIN2-TIGIT*, *CXCL8-NR3C1*, *LGALS9-HAVCR2*, *C5AR1-RPS19*, and *SPP1-PTGER4* pairs were higher in sender and receiver cells derived from tumor tissue compared with normal tissue (Figure 7d, Supplementary Figure S15b). *C5AR1* and *RPS19* were highly expressed in tumor tissue compared with normal tissue in the TCGA-CRC and TCGA-ESCA datasets (Figure 7e, Supplementary Figure S15c). *C5AR1*, *PLAUR*, *PLAU*, and *SPP1* were upregulated in late-stage compared with early-stage patients in TCGA samples (Supplementary Figure S16a–c). 

In addition, according to the above results, the correlation of L-R gene expression patterns in the same cell type showed the potential regulatory relationships that may contribute to the function of subTMEs. For example, in TAMs, gene expression of *FPR3*, *PLAUR*, and *LRP1*, the receptor genes interacting with tumor cells, was correlated with ligand genes interacting with CD8+ T cells including *C5AR1* (Figure 7f, Supplementary Figure S16d). Overall, these results together demonstrated that PLAUR was the key molecule potentially involved in macrophage polarization and C5AR1 was the key marker in TAM subtypes potentially involved in immunosuppressive TMEs by interacting with CD8+ T cells.

### 3.7. Conserved Cell-Cell Interaction Patterns and subTME Landscape in Patients in Pan-Cancer Reveal Stratification Patterns

**Figure 8:**
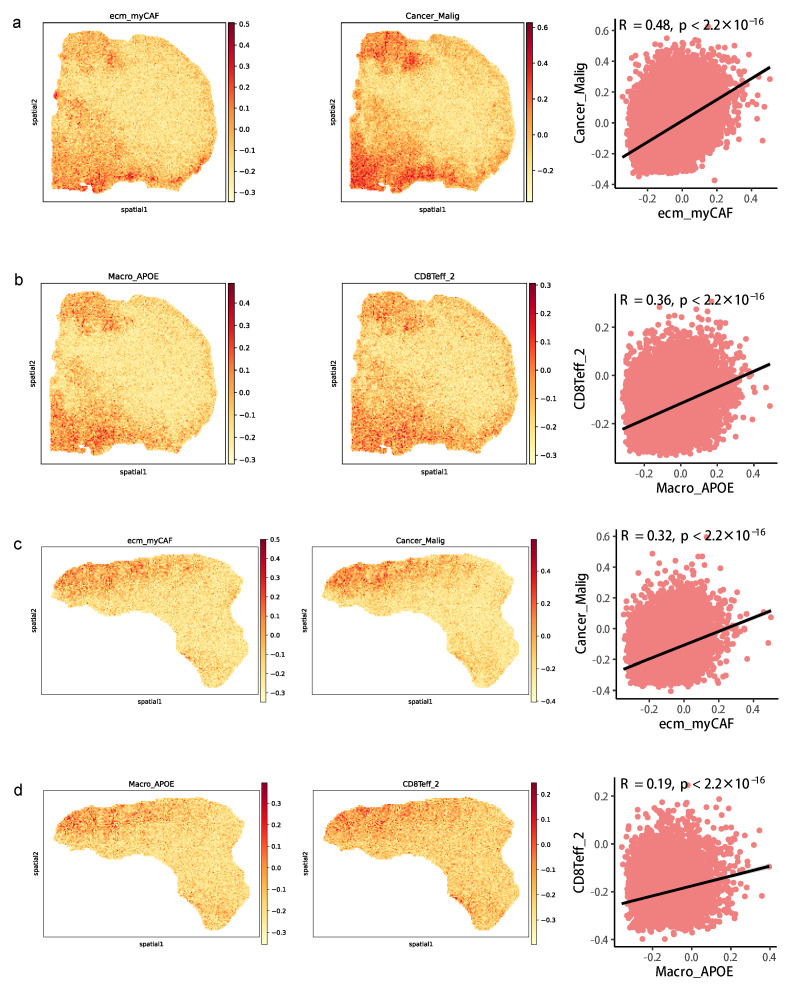
Co-localization of cell types in spatial transcriptomics data. (**a**) Spatial feature plots of signature score of ecm_myCAF (left) and Cancer_Malig (middle) in tissue sections and Spearman correlation of signature score of ecm_myCAF and Cancer_Malig (right) in CRC patient #19. (**b**) Spatial feature plots of signature score of Macro_APOE (left) and CD8Teff_2 (middle) in tissue sections and Spearman correlation of signature score of Macro_APOE and CD8Teff_2 (right) in patient #19. (**c**) The same as a but in patient #36. (**d**) The same as b but in patient #36.

We further used the spatial transcriptome (ST) data from two CRC clinical samples to detect the spatial position of the above cell types involved in CCIs within a specific subTME to verify the interaction between cell types. The data showed that the high signature scores of ecm_myCAF and Cancer_Malig were in the same spot, and their signature scores showed a significantly positive correlation in two samples, indicating that they may be co-localized in the same niche (Figure 8a,c). The same result was also observed between Macro_APOE and CD8Teff_2 (Figure 8b,d). In addition, in contrast to other spots, *COL6A1* and *SDC1* were mainly expressed in the same spot, and their expression levels were positively correlated in two samples (Supplementary Figure S17a,b). 

In addition, we considered the key molecular characteristics of the above results in the analysis of individual samples to further inspect their relationship and distribution, and we found that most of the characteristics in the individuals were consistent with the overall distribution across samples in the four cancer types in bulk data. The gene expression levels of *COL1A1-SDC1*, *COL1A1-SDC4*, *COL6A1-SDC1*, *ANXA1-FPR3*, *MDK-LRP1*, *PLAU-PLAUR*, *C5AR1-RPS19*, *CSF1-SIRPA*, *TYROBP-CD44*, *LGALS9-HAVCR2*, and *NR3C1-CXCL8* L-R pairs in each sample were ranked in ascending order of subTME scores in bulk data. Notably, the M2-polarization score and EMT score at the individual level were observed to be consistent with the change in the abundance score of subTMEs in TCGA data (Supplementary Figure S18a–c). 

## 4. Discussion

Intratumoral heterogeneity is a long-standing obstacle to defining the precise contributions of the TME to cancer progression. Our study integrates single-cell analyses with clinical information and systematically reveals how TME heterogeneity is a part of factors driving tumor progression in pan-cancer. Specifically, we discovered two tumor-promoting microenvironmental states distinct from precancerous states in tissues. The tumor-promoting subsets are immunosuppression- and stroma-related subTMEs, while the other two are immune-infiltration- and precancerous epithelial enrichment-related subTMEs, which display variance in cell type composition, cell phenotype, and cell-cell interaction patterns. 

The current study reveals that the stroma-related subTME is abnormally active in angiogenesis, active matrix remodeling, and tumor invasion potential. The immunosuppression-related subTME contains anti-inflammatory macrophages and exhausted CD8+ T-cell infiltration, which frequently coexist intratumorally with the stroma-related subTME and potentially support tumor progression. Notably, *PDCD1* and *TGFB1*, as immunosuppressive factors [35,36], are highly expressed in subTME-IS. The immune-infiltration-related subTME is vascularized and abundant with anti-tumor effector cell infiltration. The precancerous epithelial enrichment-related subTME mainly includes non-malignant epithelial cells. Accordingly, tumors benefit from the concomitant presence of stroma- and immunosuppression-related subTMEs, resulting in tumor progression. Notably, there is a significant difference in T-lymphocyte infiltration as well as immunosuppressive cell levels across cancer types. As shown in the results (Supplementary Table S2), BC and PDAC have lower levels of T-cell infiltration, which is consistent with previous studies [37,38] concerning heterogeneity between tumor types, and these tumor types have been called “cold” tumors due to the low level of immune cell infiltration and high failure rate of immunotherapy. For example, the complex TME of pancreatic cancer, consisting of an abundance of blood vessels, fibroblasts, and pancreatic stellate cells, has been revealed to have less immune cell infiltration and diminished immunosuppressive features [39]. These cancer types and samples, with low levels of immune cell infiltration, are identified as immunologically quiet [40] and might be suitable for chemotherapy or targeted therapy to eliminate tumor cells and facilitate T-cell infiltration [41]. CAFs represent a major component of the TME and suppress the infiltration of CD8+ T cells into the tumor site in breast cancer; targeting CAF subsets via genetic deletion facilitates CD8+ T-cell infiltration and enhances sensitivity to immunotherapy [42], further enhancing clinical outcomes [43]. Aside from the cancer types studied in the current study, gastrointestinal stromal tumors (GISTs), as the most common mesenchymal tumors, were found to have less effective cytotoxic T lymphocytes but a higher number of monocyte-derived cells, especially the M2-like TAMs in the local immunosuppressive TME [44,45]. Particularly, the infiltration level of M2-like TAMs negatively correlates with T-cell presence (CD4+T and CD8+T together), supporting the existence of the immunosuppressive effect of M2-like TAMs on immune infiltration at the tumor site [46]. In a single-cell study, results showed that there was a high level of expansion of T cells (35.2% and 56.7% among high- and low-risk patients); however, the cells were highly exhausted in the samples, indicating that the pro-inflammation and cell-killing effects of T cells are suppressed in GISTs [47]. Understanding the “cold” immune microenvironment has been an ongoing theme of research, and there has been an increase in research aiming to provide appropriate combination treatment methods and guidance for improving the anti-tumor immune response [48]. Therefore, further research is needed to classify cancer types as well as cancer patients with low immune infiltration to find more suitable and effective treatment methods [48,49]. 

It is observed that the main components in the stroma-related subTME, including ecm-myCAF, wound-myCAF, and angiogenic-End, frequently interact with tumor cells and together establish coordinated phenotypes with a pEMT phenotype and the behavior of tumor cells. In particular, genes encoding collagen, including *COL1A1*, *COL1A2*, *COL6A1*, and *COL6A3*, are upregulated in tumor tissue and also highly expressed in late-stage cancer patients; they are predicted to interact with *SDC1* and *SDC4* in tumor cells to promote the pEMT phenotype of tumors. Altered expression of *COL1A1* has been observed in numerous cancer types, including CRC [50], ovarian cancer (OV) [50], gastric cancer (GC) [50], melanoma [50], and glioblastoma [50], and promotes the proliferative and invasive ability of tumor cells. However, *COL6A2* and *COL6A2* are currently less studied. SDC1, one of the heparan sulfate proteoglycans, is involved in the metastasis of BC [50], prostate cancer [50], endometrial cancer [50], and oral squamous cell carcinoma [50]. In addition, one study reveals that SDC1 is a critical mediator of micropinocytosis and tumor growth and is a potential target in pancreatic cancer [50]. SDC4 is involved in regulating tumor cell migration in both hepatocellular carcinoma [50] and BC [50]. The *COL1A1*-*SDC1*, *COL6A2*-*SDC1*, and *COL6A3*-*SDC1* gene pairs are revealed to be highly expressed in late-stage patients compared with early-stage patients in the current study, but the molecular mechanisms remain unknown. Thus, although these molecules serve as separate targets, our data suggest interactions between *COL1A1*-*SDC1*, *COL6A2*-*SDC1*, and *COL6A3*-*SDC1* may serve as a target for therapeutic intervention in pan-cancer by intervention in the binding sites of two molecules. 

TAMs including Macro_APOE, Macro_ACP5, and Macro_SPP1 are the main components in the immunosuppression-related subTME. Our findings reveal that Macro_APOE harbors the anti-inflammatory and M2-macrophage polarization phenotype resulting in inhibiting the infiltration and activation anti-tumor function of immune cells. The transcriptional profile of subTME-IS harboring exhausted CD8+ T cells reveals a dysregulated anti-tumor phenotype, including higher levels of checkpoint molecules and T-cell exhaustion scores compared to other subTMEs. *CXCL8*-*NR3C1*, *SPP1*-*PTGER4*, and *C5AR1*-*RPS19* are L-R gene pairs between TAMs and CD8+ T cells in the current study. *NR3C1*, named glucocorticoid receptor gene, is involved in regulating immune function. One recent study shows that NR3C1 signaling affects the function of CD8+ T cells and shows a gradient of increasing *NR3C1* expression and signaling from naïve to dysfunctional CD8+ T cells [50]. *NR3C1* is revealed to be associated with the failure of checkpoint blockade [50], and the loss of *NR3C1* potentiates the response to checkpoint blockade, suggesting inhibiting NR3C1 is a potential strategy to improve anti-tumor immune responses in combination with other treatments. CXCL18 is predicted to interact with NR3C1 in our data, suggesting that *CXCL18* and *CXCL18*-expressing TAMs are novel moderators of dysfunctional CD8+ T cells. PTGER4 is involved in modulating the immune TME, and inhibiting PTGER4 using an antagonist could alleviate the immunosuppressive microenvironment and further enhance the proliferation and anticancer functions of T cells [50]. SPP1-PTGER4 interaction is also revealed in liver cancer [50]. C5AR1 expression in myeloid-derived suppressor cells promotes cancer metastasis [50] and the infiltration of immunosuppressive leukocyte populations into the TME [50]. Inhibiting C5AR1 enhances chemotherapy response by involving the suppression of CD8+ T-cell cytotoxicity, and a study suggests C5AR1-dependent signaling as an important immunomodulatory program for combinatorial cancer immunotherapy [50]. There are few studies on C5AR1-RPS19 pairs between TAMs and CD8+ T cells. One study reveals that RPS19 expression in tumor cells promotes infiltration of regulatory T cells and reduces infiltration of CD8+ T cells into tumors by interaction with C5AR1 in myeloid-derived suppressor cells [50]. Blocking C5AR1-RPS19 interaction decreases RPS19-mediated immunosuppression and impairs tumor growth in a breast cancer model [50]. Our results show that *C5AR1* is specially expressed in TAMs and its expression is correlated with the M2-polarization score, indicating C5AR1+ TAMs may be potential targets for alleviating the immunosuppressive microenvironment and further improving the anti-tumor ability of immune cells. 

Limitations of this study should be noted to avoid overinterpretation. First, although we profiled the ubiquitous characteristics and functions of different TME subtypes at the single-cell resolution, additional experimental and clinical efforts are needed to determine the pro-tumor roles of these functional subtypes, to establish causality between anti-inflammatory TAMs as well as tumor cells and ECM-related CAFs within a subTME, and further to determine whether inhibiting these circuits would effectively reinvigorate an anti-tumor immune response and inhibit tumor invasion. Second, we only demonstrated the potential cellular interaction between cells within subTMEs mainly through bioinformatic approaches in single-cell and ST data. However, verification through multiple immunofluorescences with specific markers of clinical samples is lacking and should be explored in the future. Third, the samples included in our study were from public databases; we did not produce the sequencing data ourselves or verify the analysis results. Fourth, there were no differences in TME subtype scores between low-grade and high-grade tumors; it remains to be determined which CCI signals within a specific subTME determine the tumor development and progression, and a larger sample cohort and in-depth analysis will be further needed in the future. 

## 5. Conclusions

**Figure 9:**
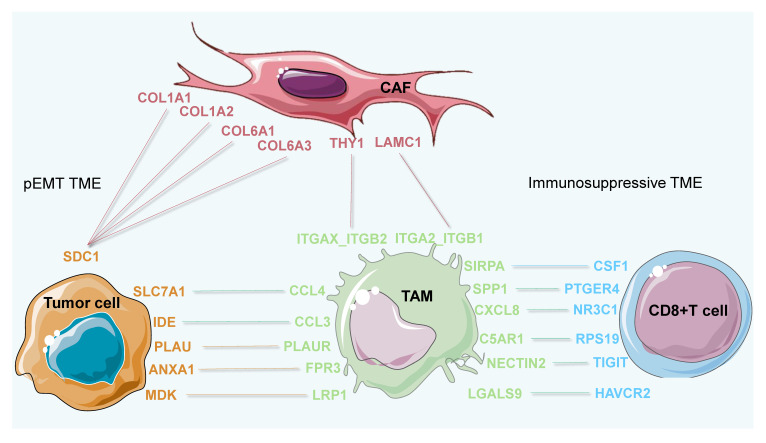
Schema of cellular interactions between cell types of interest within both subTME-MRM and subTME-IS.

Together, these findings suggest the potential interactions and L-R pairs between CAFs and tumor cells as well as among tumor cells, TAMs, and CD8+ T cells, which might contribute to the state and function of subTME-MRM and subTME-IS, respectively. We propose that, mechanistically, CAFs might cause tumor cells to exhibit the proliferative and pEMT states via L-R interactions including COL1A1-SDC1, COL6A2-SDC1, and COL6A3-SDC1. The interaction between tumor cell subpopulations and TAMs (for example, via PLAU-PLAUR) potentially contributes to the reprogramming of TAMs into the immunosuppressive phenotype. These TAMs might in turn suppress CD8+ T-cell function and further potentially exacerbate the malignant phenotype of tumor cells (Figure 9). Collectively, although further experimental verification is warranted to establish the functional role of each subTME and the biological interaction mechanisms of these key CCI pairs, our pan-cancer study on subTMEs may facilitate accurate subTME-targeted classification, therapy target development, and applications in the future.

## Data Availability

All data used in this study are publicly available as described in the Methods section within the article and its supplementary data files. The main source codes for the analysis and visualization of this study are available at the GitHub repository: https://github.com/Dulab2020/sc-analysis/tree/main/Analysis/CCI (accessed on 1 May 2023).

## Supplementary Materials

The following supporting information can be downloaded at: https://www.mdpi.com/article/10.3390/biomedicines11113057/s1, Figure S1. TME subtypes across cancers. a. UMAP plots of cells from normal and tumor tissue of ESCA patients, showing 6 clusters indicating cell type, 2 clusters indicating cells derived from tumor and normal tissues. Each cluster is shown in a different color. b. The same as shown in a but in PDAC patients. c. The three cellular modules on the basis of correlations of cell subclusters from tumors with positive (Spearman correlation; correlation coefficient r > 0.3 and FDR < 0.05, in red), negative (r < −0.3 and FDR < 0.05, in blue), or non-significant (white) pairwise correlation for infiltration in ESCA samples. d. The four cellular modules on the basis of correlations of cell subclusters from tumors in PDAC samples. e,f. subTME score in normal and tumor groups in ESCA and PDAC. *** *p* < 0.001, ** *p* < 0.01, * *p* < 0.05, Wilcoxon tests. Figure S2. TME subtype score across cancers. a. subTME score in normal and tumor sample groups in BC, CRC, and ESCA TCGA data. *** *p* < 0.001, Wilcoxon tests. b. The five cellular modules on the basis of correlations of cell subclusters from tumors with positive (Spearman correlation; correlation coefficient r > 0.3 and FDR < 0.05, in red), negative (r < −0.3 and FDR < 0.05, in blue), or non-significant (white) pairwise correlation for infiltration in LC samples. d. The four cellular modules on the basis of correlations of cell subclusters from tumors in RCC samples. e,f. subTME score in normal and tumor sample groups in LC and RCC. *** *p* < 0.001, ** *p* < 0.01, * *p* < 0.05, Wilcoxon tests. Figure S3. Definition of TME subtypes. a. Boxplot showing the myeloid cells’ traffic score among subTMEs in BC and ESCA, *** *p* < 0.001, * *p* < 0.05, Kruskal–Wallis test. b. Boxplot showing the epithelial cells’ score among subTMEs in ESCA and PDAC. c. Scatterplot showing the Spearman correlation between the score of angiogenesis, matrix remodeling, EMT, and tumor proliferation and subTME-MRM score in ESCA. d. The same as c but in PDAC. e. The same as c but in BC. Figure S4. a. Heatmap of cytokine, chemokine, and cytotoxic mediator gene expression between different major cell types. b. Violin chart of PDCD1 and CD247 expression across cancer types. *** *p* < 0.001, Wilcoxon tests. Figure S5. Gene expression of cytokines, chemokines, and cytotoxic mediators in epithelial cells from normal and tumor tissues across four cancer types. *** *p* < 0.001, ** *p* < 0.01, * *p* < 0.05, Wilcoxon tests. Figure S6. Gene expression of cytokines, chemokines, and cytotoxic mediators in lymphocytes and natural killer cells from normal and tumor tissues across four cancer types. *** *p* < 0.001, ** *p* < 0.01, * *p* < 0.05, Wilcoxon tests. Figure S7. Gene expression of cytokines, chemokines, and cytotoxic mediators in myeloid cells from normal and tumor tissues across four cancer types. *** *p* < 0.001, ** *p* < 0.01, * *p* < 0.05, Wilcoxon tests. Figure S8. Gene expression of cytokines, chemokines, and cytotoxic mediators in fibroblasts from normal and tumor tissues across four cancer types. *** *p* < 0.001, ** *p* < 0.01, * *p* < 0.05, Wilcoxon tests. Figure S9. Gene expression of cytokines, chemokines, and cytotoxic mediators in endothelial cells from normal and tumor tissues across four cancer types. *** *p* < 0.001, ** *p* < 0.01, * *p* < 0.05, Wilcoxon tests. Figure S10. Boxplot showing the gene expression of cytokines, chemokines, and cytotoxic mediators among subTMEs across four cancer types, *** *p* < 0.001, * *p* < 0.05, Kruskal–Wallis test. Figure S11. KM survival analysis, risk score assessment by the subTME gene markers and time-dependent ROC curves in BC and ESCA datasets. a. KM survival analysis of high- and low-risk samples. b. Relationship between the survival status/risk score rank and survival time/risk score rank. c. Time-dependent ROC curve for overall survival of the BC datasets. d–f. The same as a–c but in ESCA datasets. Figure S12. Cell-cell interaction across cell subclusters. a–c. Heatmap showing the number of interactions between cell types within subTME-MRM in BC, ESCA, PDAC. d-g. Functional enrichment analysis of ligand–receptor genes in each subTME in BC, CRC, ESCA, and PDAC. Figure S13. Cell–cell interaction gene pairs across cell subclusters. a–c. Enrichment of selected ligand–receptor interactions for the cell-type pairs within subTME-IS in BC, ESCA, and PDAC. d–f. Enrichment of selected ligand–receptor interactions for the cell-type pairs within subTME-MRM in BC, CRC, and PDAC; the bubbles shown in the figure indicate *p* < 0.05; the color of the bubbles indicates the level of mean gene expression of ligand–receptor. Figure S14. EMT phenotype of tumor cells is regulated by tumor-enriched molecules expressed and secreted by fibroblasts. a. Scatterplot showing the Spearman correlation of the gene expression in fibroblasts and cell proportion of tumor cells in BC. b. Boxplot showing the different expression of ligand or receptor genes between different stages of patients. *** *p* < 0.001, ** *p* < 0.01, * *p* < 0.05, Wilcoxon tests. Figure S15. Immunosuppressive microenvironment regulated by TAMs. a. Boxplot showing the exhaustion score between T cells in CRC, *** *p* < 0.001, Kruskal–Wallis test. b. Bubble chart showing the gene expression of ligand–receptor pairs derived from tumor cells and TAMs or TAMs and CD8+ T cells, respectively, between normal and tumor samples in single-cell data in BC, CRC, and ESCA; the color of the bubbles indicates the level of gene expression of a ligand or receptor in sender or receiver cells; the bubbles shown in the figure indicate *p* < 0.05, Wilcoxon tests. c. Boxplot showing the gene expression of ligand–receptor pairs between normal and tumor samples in TCGA-BC and TCGA-CRC data, *** *p* < 0.001, * *p* < 0.05, Wilcoxon tests. Figure S16. Ligand-receptor interactions between TAMs and T cells. a–c. Boxplot showing the different expression of ligand or receptor genes between different stages of patients, ** *p* < 0.01, * *p* < 0.05, Wilcoxon tests. d. Heatmap showing the Spearman correlation between genes, *p* < 0.05. Figure S17. Co-localization of ligand–receptor genes in spatial transcriptomics data. a. Spatial feature plots of gene expression of COL6A1 (left) and SDC1 (middle) and Spearman correlation between COL6A1 and SDC1 (right) in patient #19. b. Spatial feature plots of gene expression of COL6A1 (left) and SDC1 (middle) and Spearman correlation between COL6A1 and SDC1 (right) in patient #36. Figure S18. Overview of CCI gene pairs in different subTMEs. a–d. Molecular characteristics of different cell types and CCI gene pairs across samples in BC, ESCA, PDAC, and CRC TCGA data. Table S1. The resolution of subclusters in single-cell data. Table S2. Cell types, cell number, and cell proportion in each patient across four cancer types. Table S3. Gene signature for Hallmark subTME used in TCGA data and single-cell data. Table S4. Proportion of cell types and subTME score in each patient across cancer types. Table S5. The cytokines, chemokines, and cytotoxic mediators used in the current study. Table S6. Construction of subTME-based risk model. Table S7. Correlation between gene expression in ligand cells and the proportion of receptor cells across cancer types.

## Abbreviations

AUC	area under the curve
BC	breast cancer
CAF	cancer-associated fibroblast
Cancer_Malig	cancer malignant cell
CCI	Cell-cell interaction
CD4+T	CD4+ T lymphocytes
CD8+T	CD8+ T lymphocytes
cDC	conventional DC
CRC	colorectal cancer
DC	dendritic cell
DEGs	differentially expressed genes
ECM	extracellular matrix
EMT	epithelial-to-mesenchymal transition
End	endothelial cells
ESCA	esophageal cancer
GIST	gastrointestinal stromal tumor
KM	Kaplan–Meier
LC	lung cancer
L-R	ligand–receptor
Macro	macrophage
myCAF	myofibroblast
NK	natural killer
OV	ovarian cancer
PC	principal component
PDAC	pancreatic ductal adenocarcinoma
pEMT	partial epithelial-to-mesenchymal transition
RCC	renal cell carcinoma
ROC	receiver operating characteristic
scRNA	single-cell RNA
ssGSEA	single sample gene set enrichment analysis
ST	spatial transcriptome
subTME	tumor microenvironment subtype
subTME-ICI	immune cell infiltration tumor microenvironment subtype
subTME-IS	immune-suppressive tumor microenvironment subtype
subTME-MRM	matrix remodeling with malignant cells tumor microenvironment subtype
subTME-PSE	precancerous state of epithelial cells tumor microenvironment subtype
TAM	tumor-associated macrophage
Teff	T lymphocyte effector
Tex	T lymphocyte exhaustion
TME	tumor microenvironment
Treg	T regulatory
