# Supplementary figures and images for: Intercellular Molecular Crosstalk Networks within Invasive and Immunosuppressive Tumor Microenvironment Subtypes Associated with Clinical Outcomes in Four Cancer Types
Source: Biomedicines. 2023 Nov 14;11(11):3057. doi: 10.3390/biomedicines11113057 (PMC10669098; doi:10.3390/biomedicines11113057)

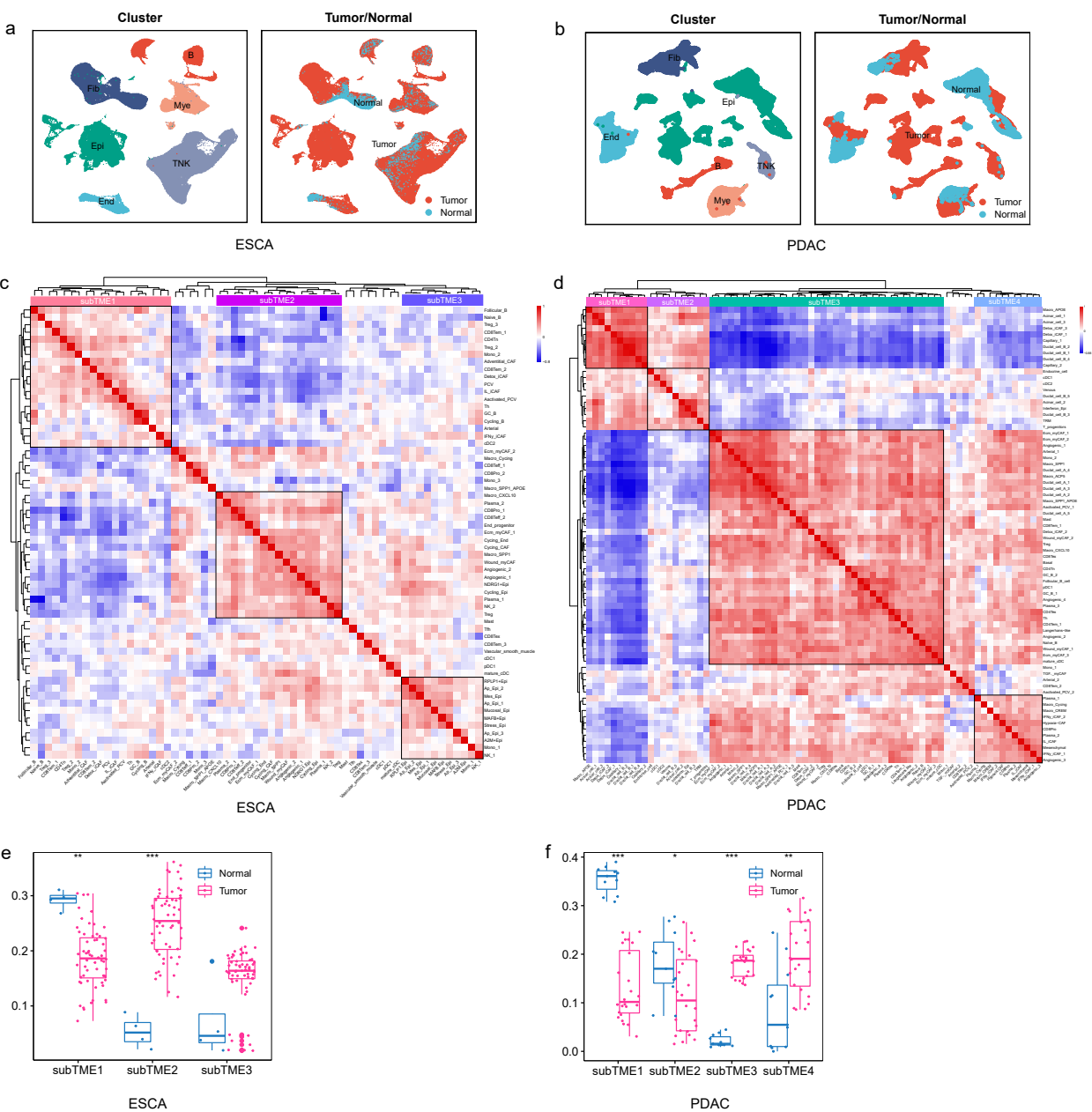

Supplement: Supplementary file 1 [file biomedicines-11-03057-s001.zip › Supplementary Figure S1.pdf]

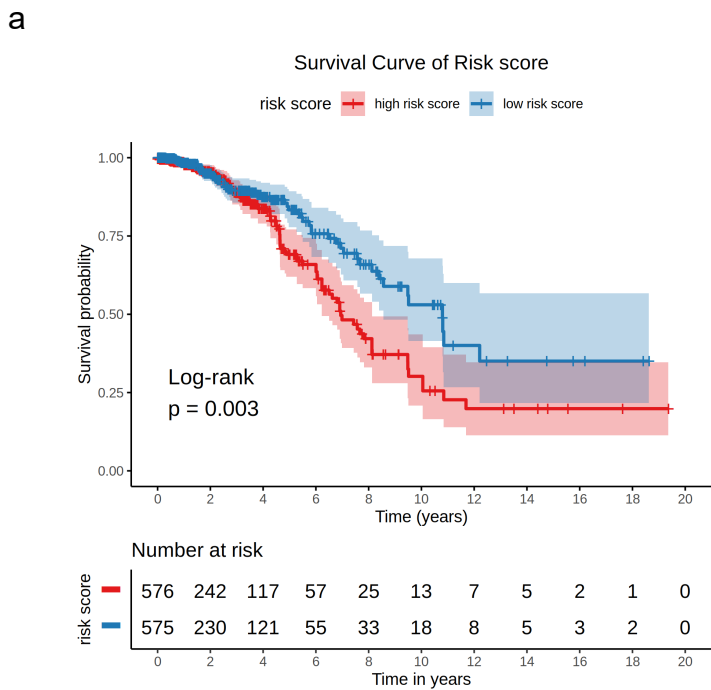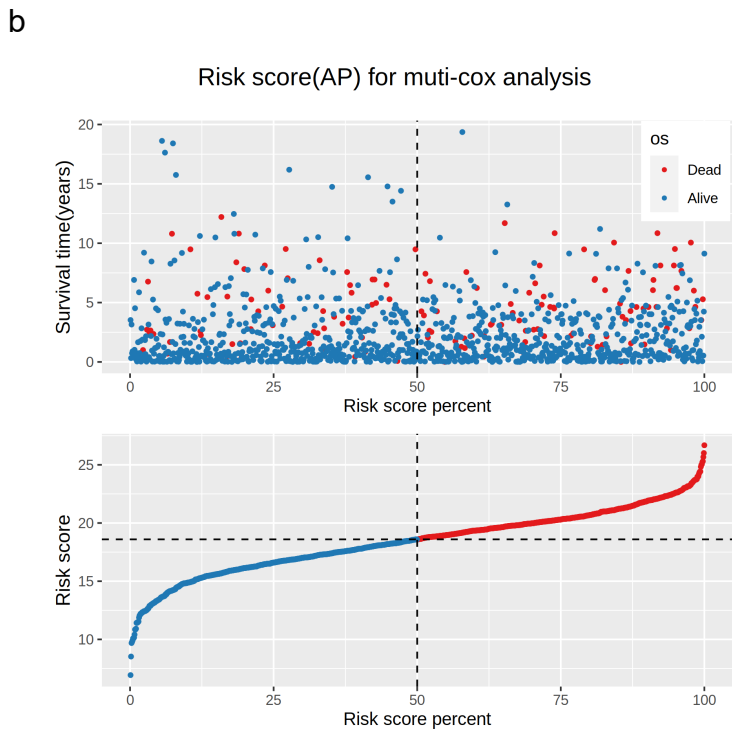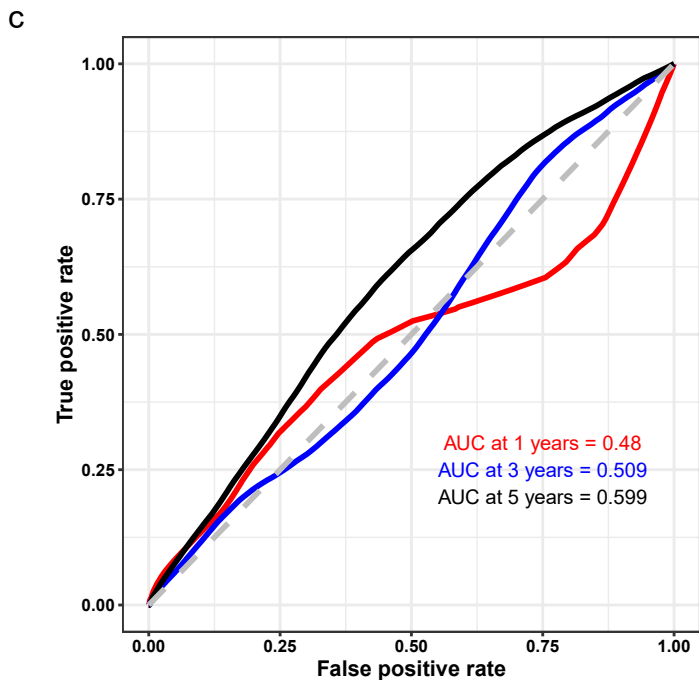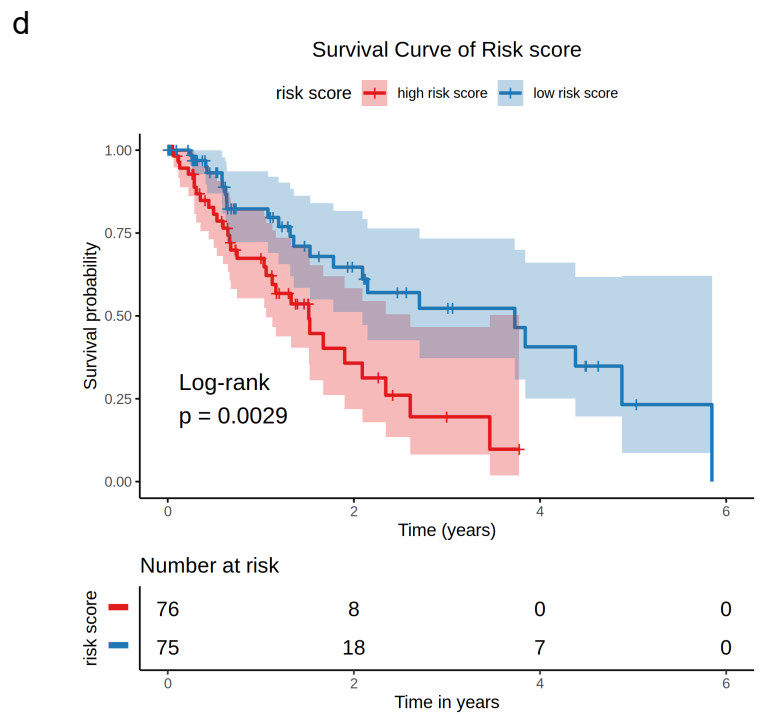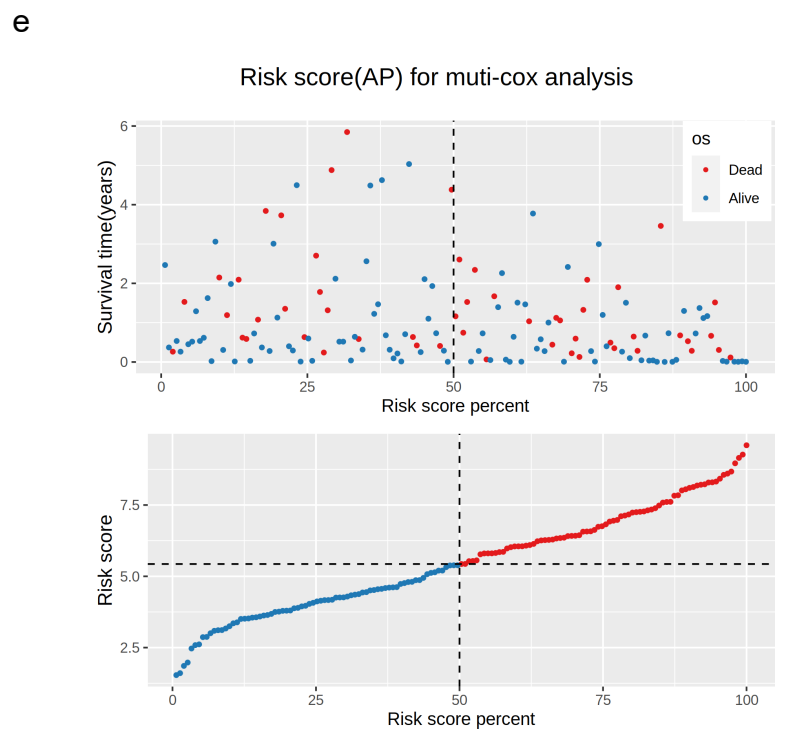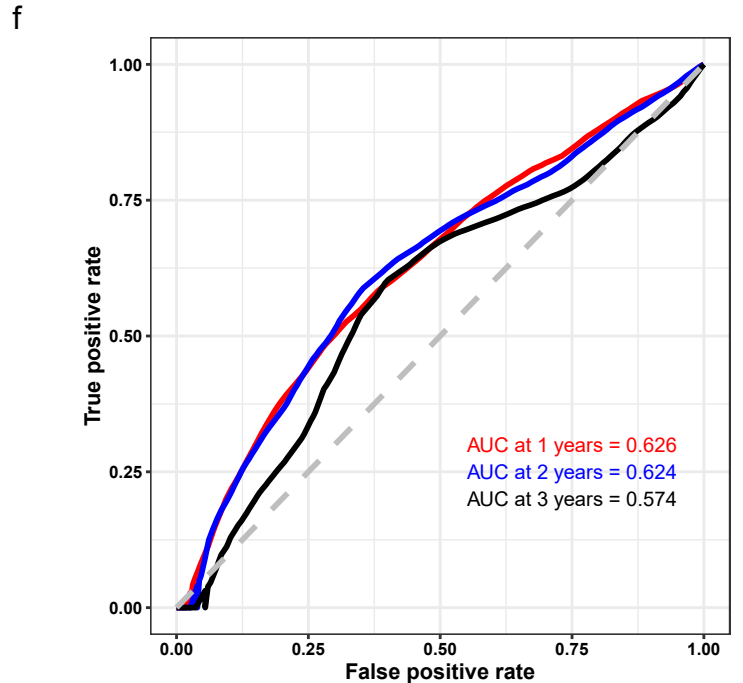

Supplement: Supplementary file 1 [file biomedicines-11-03057-s001.zip › Supplementary Figure S11.pdf]

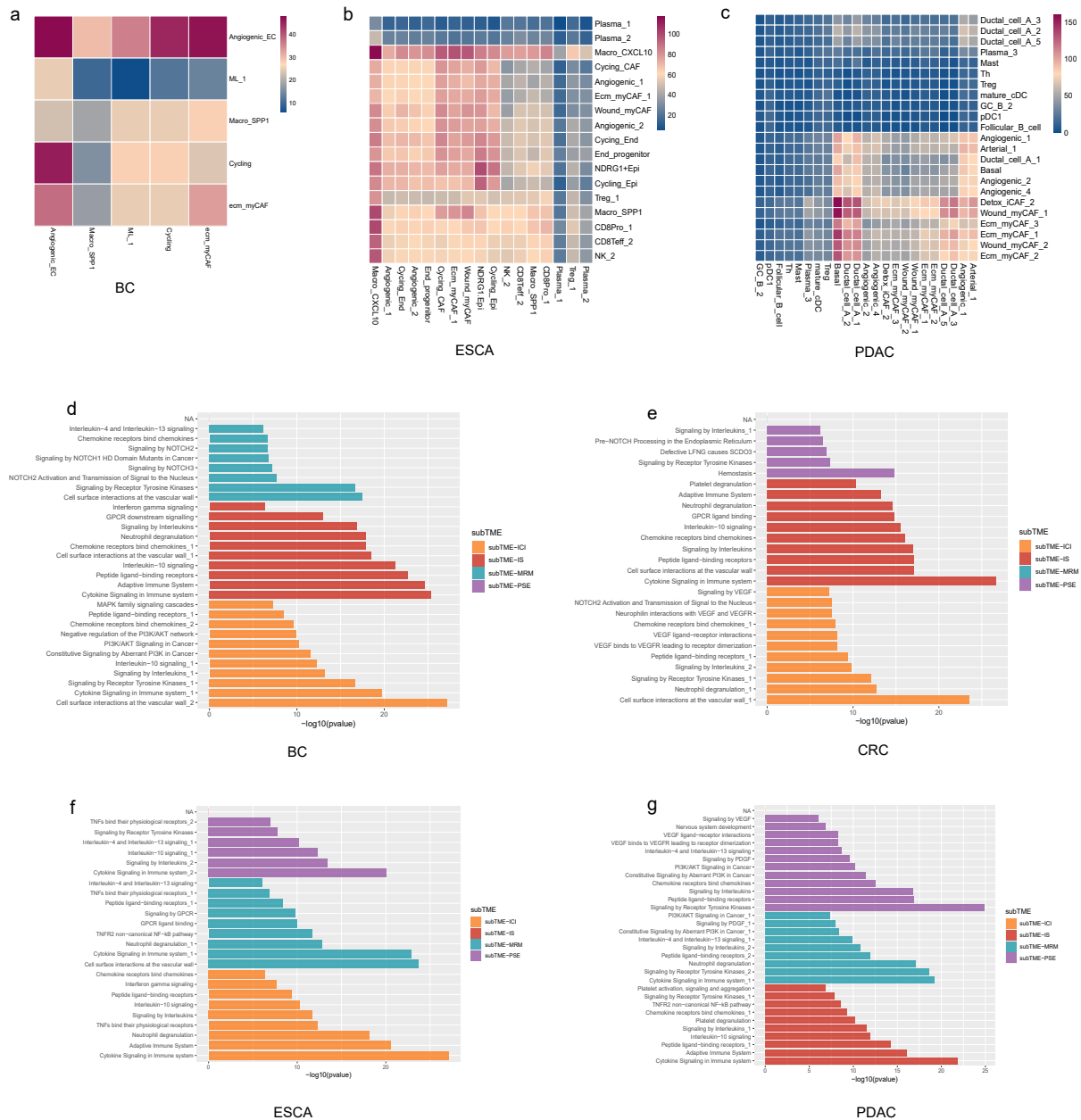

Supplement: Supplementary file 1 [file biomedicines-11-03057-s001.zip › Supplementary Figure S12.pdf]

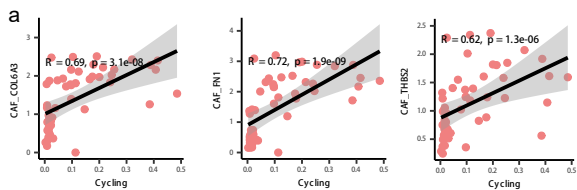

BC

CRC

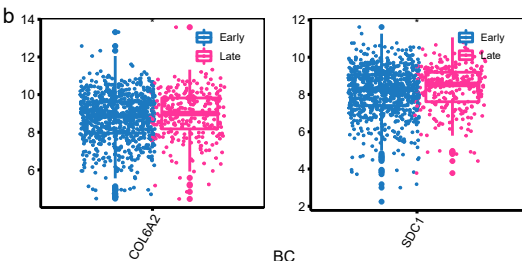

BC

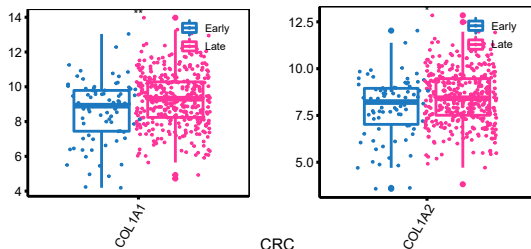

CRC

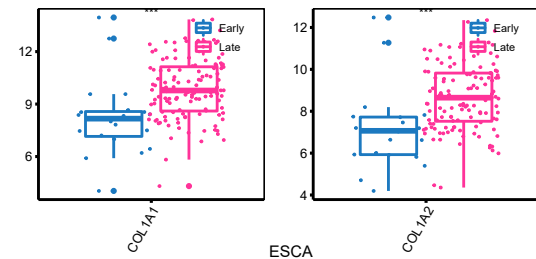

ESCA

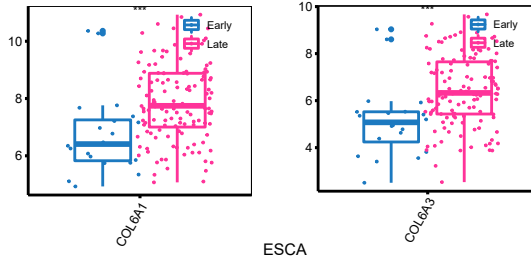

ESCA

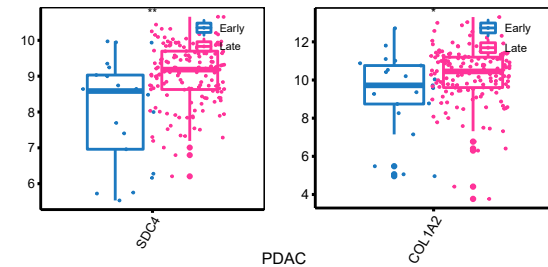

PDAC

Supplement: Supplementary file 1 [file biomedicines-11-03057-s001.zip › Supplementary Figure S14.pdf]

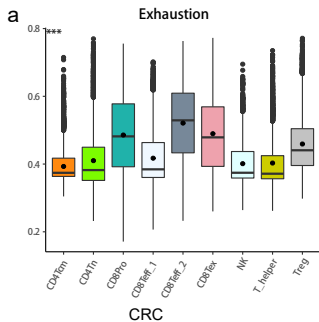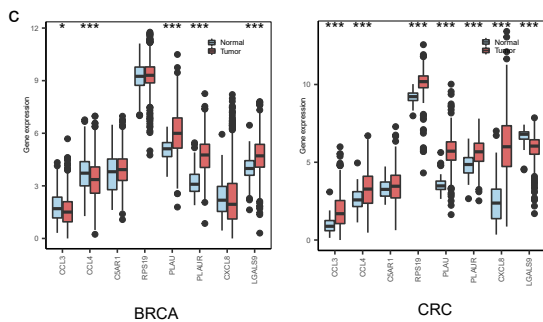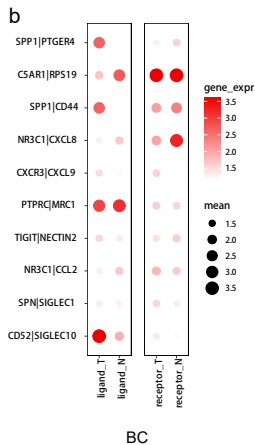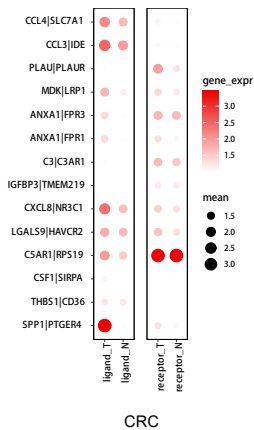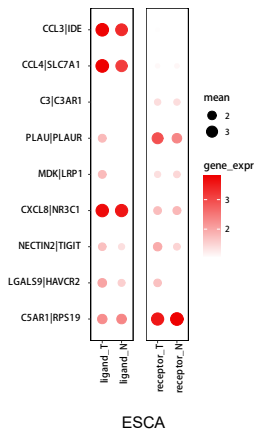

Supplement: Supplementary file 1 [file biomedicines-11-03057-s001.zip › Supplementary Figure S15.pdf]

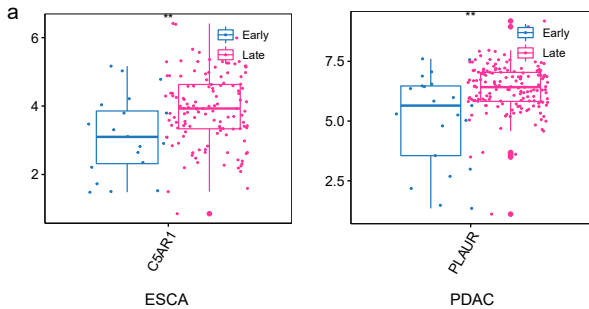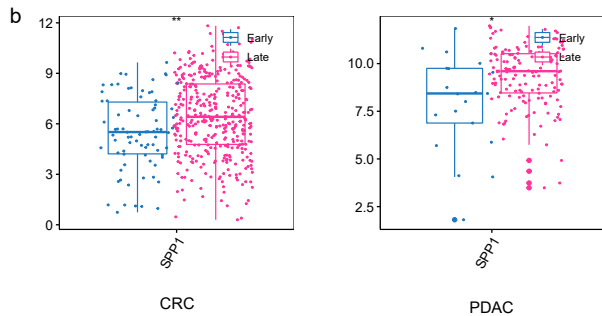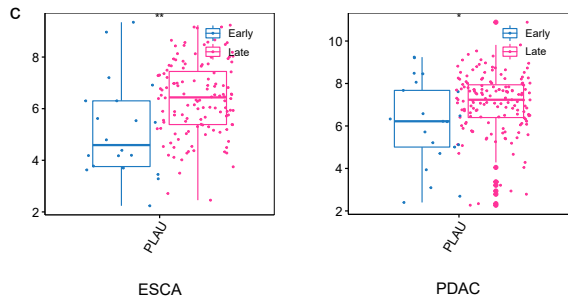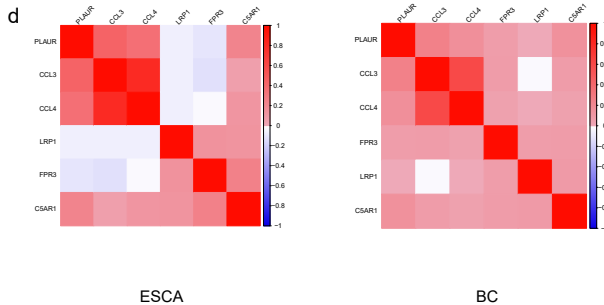

Supplement: Supplementary file 1 [file biomedicines-11-03057-s001.zip › Supplementary Figure S16.pdf]

a

COL6A1

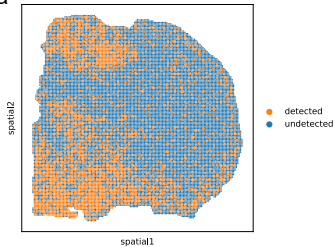

SDC1

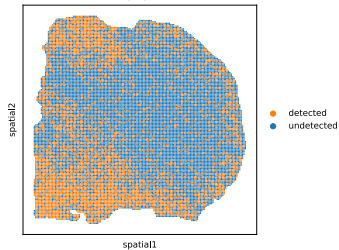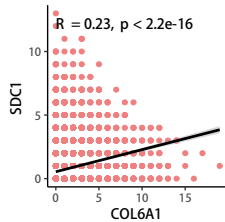

b

COL6A1

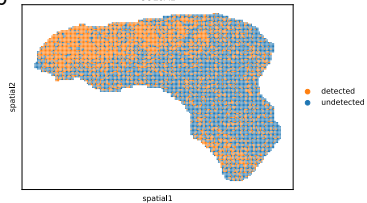

SDC1

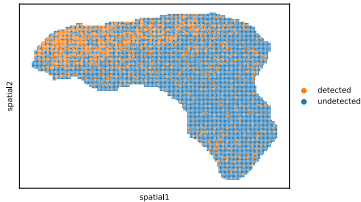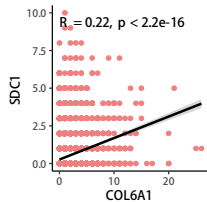

Supplement: Supplementary file 1 [file biomedicines-11-03057-s001.zip › Supplementary Figure S17.pdf]

a

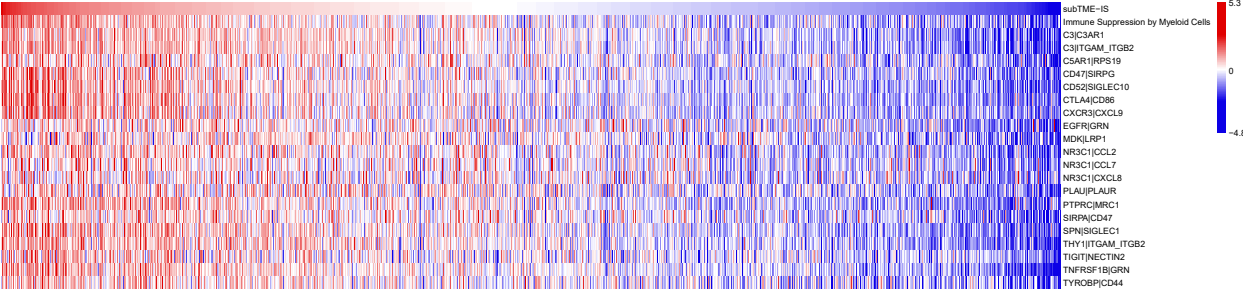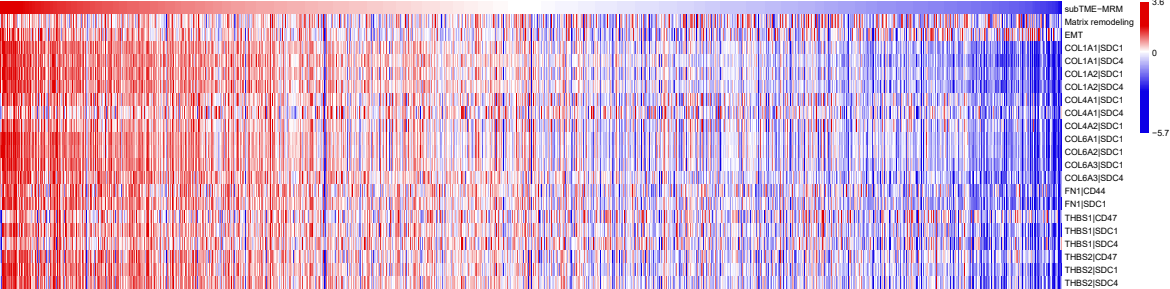

BC

b

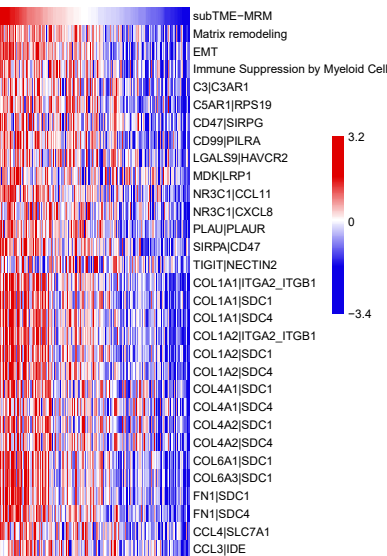

ESCA

c

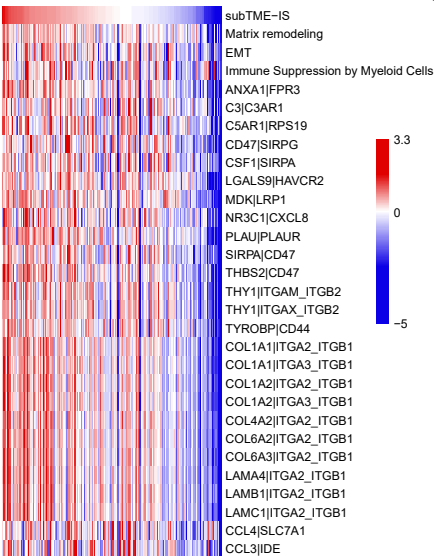

PDAC

d

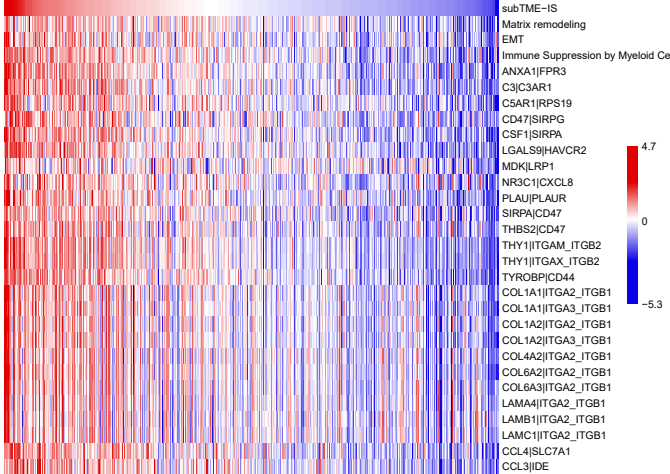

CRC

Supplement: Supplementary file 1 [file biomedicines-11-03057-s001.zip › Supplementary Figure S18.pdf]

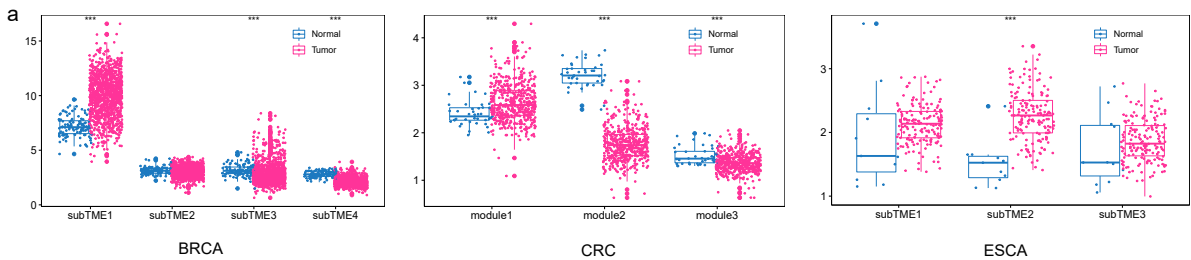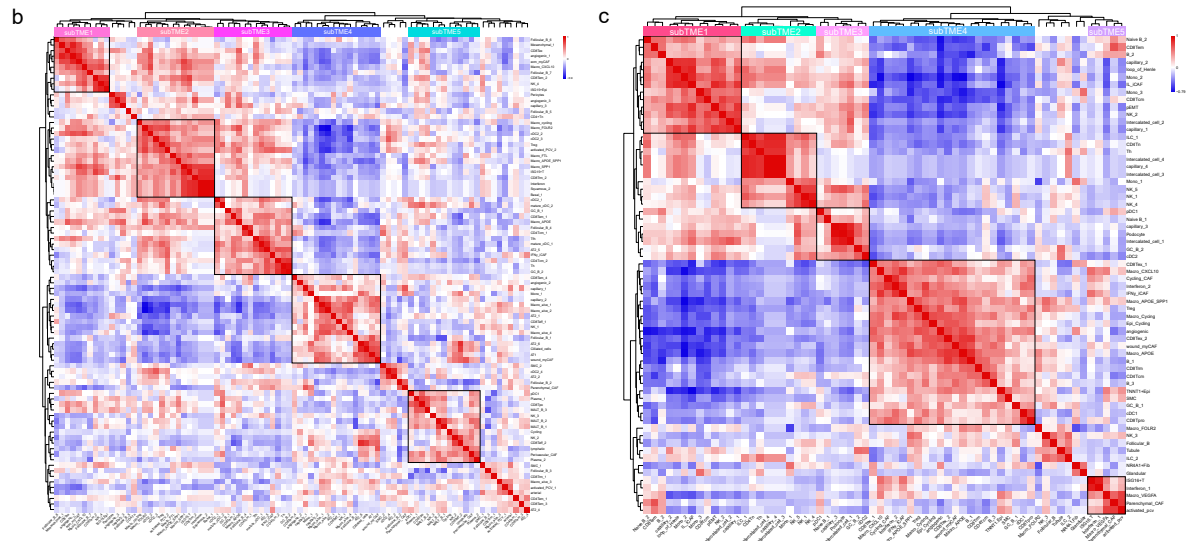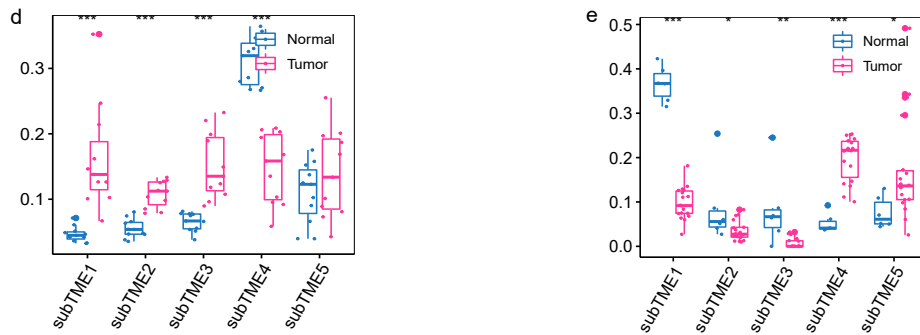

Supplement: Supplementary file 1 [file biomedicines-11-03057-s001.zip › Supplementary Figure S2.pdf]

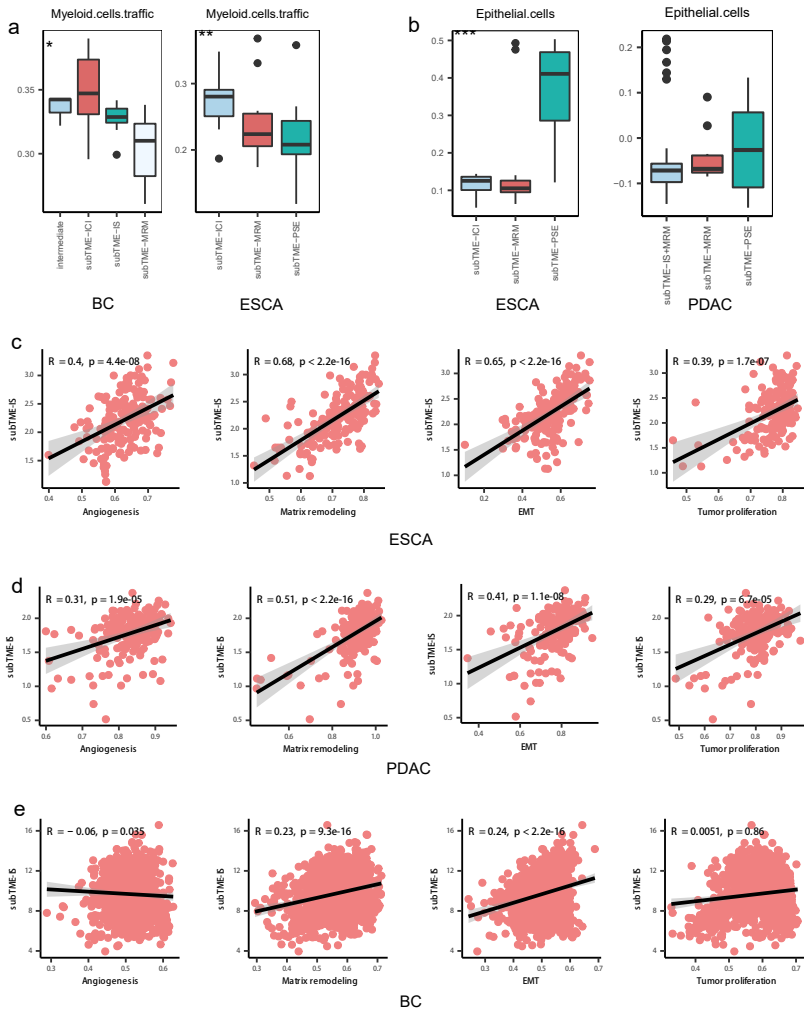

Supplement: Supplementary file 1 [file biomedicines-11-03057-s001.zip › Supplementary Figure S3.pdf]

a

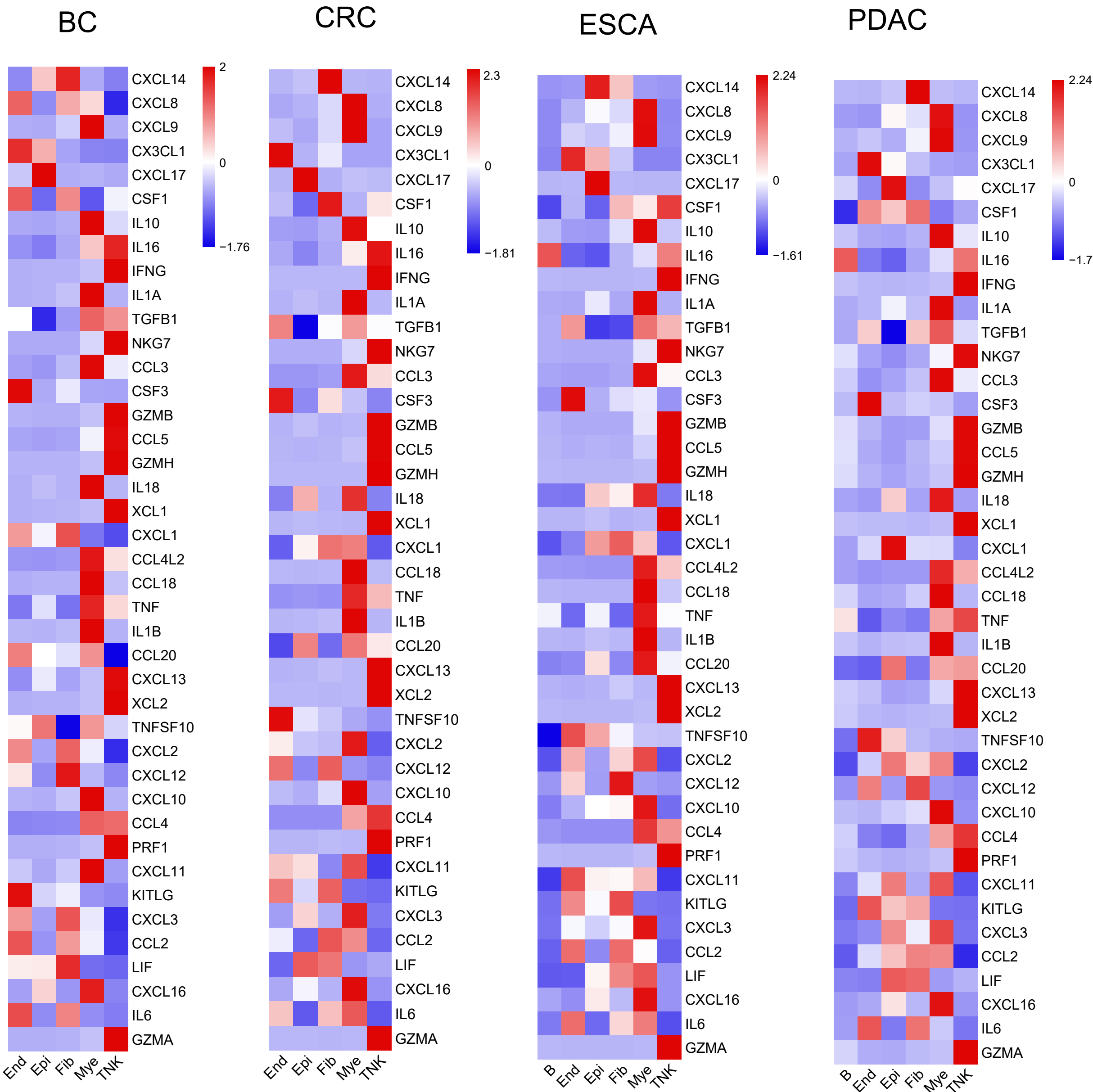

b

BC

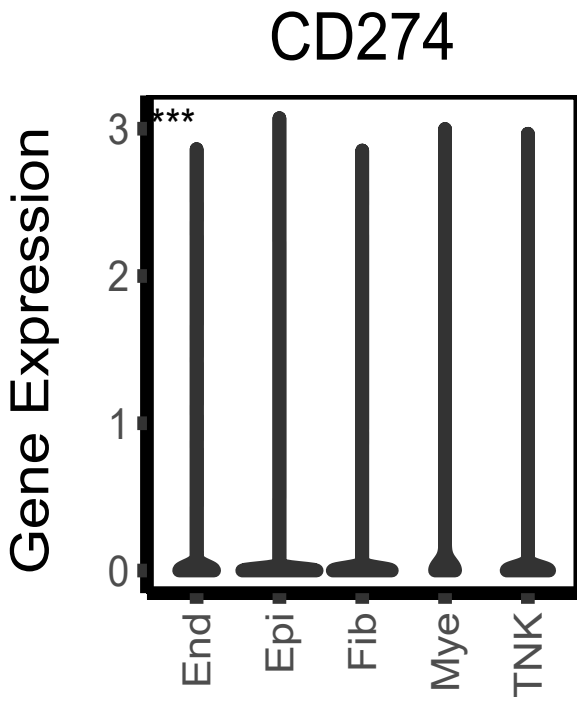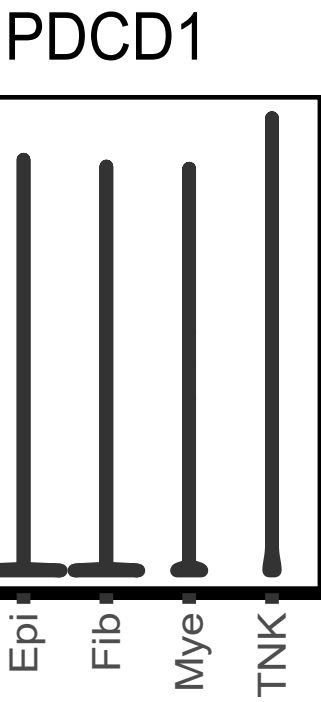

CRC

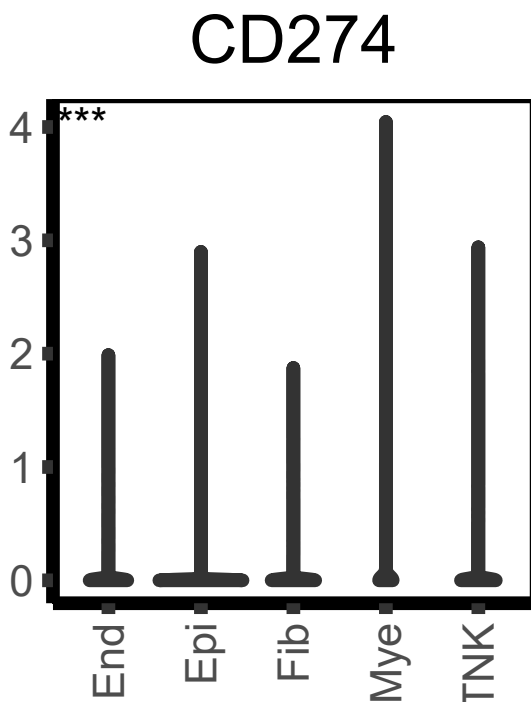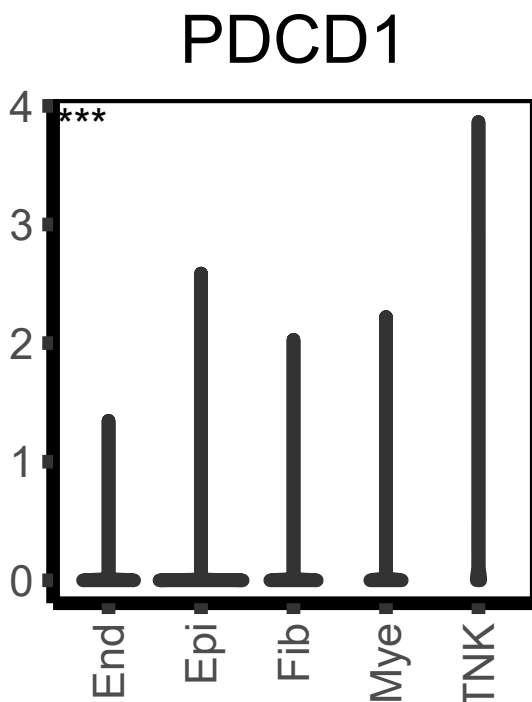

ESCA

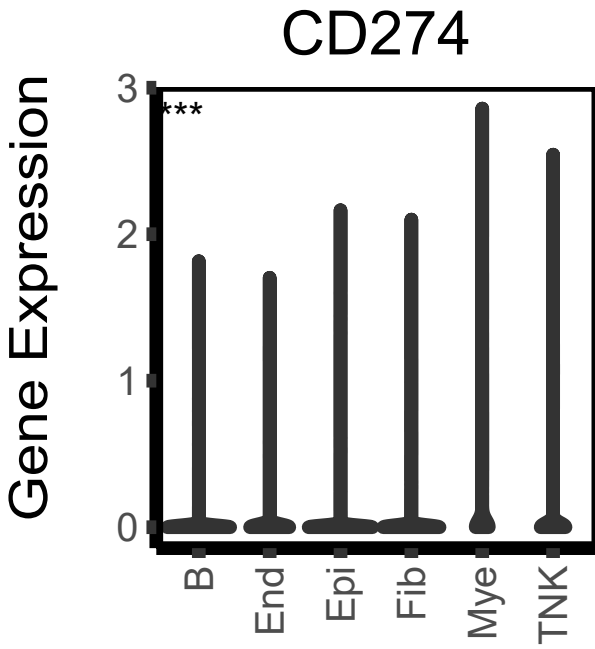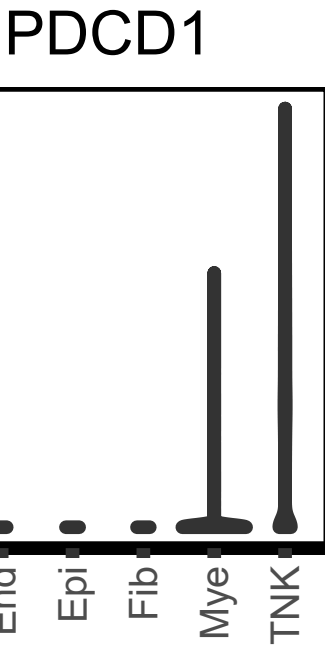

PDAC

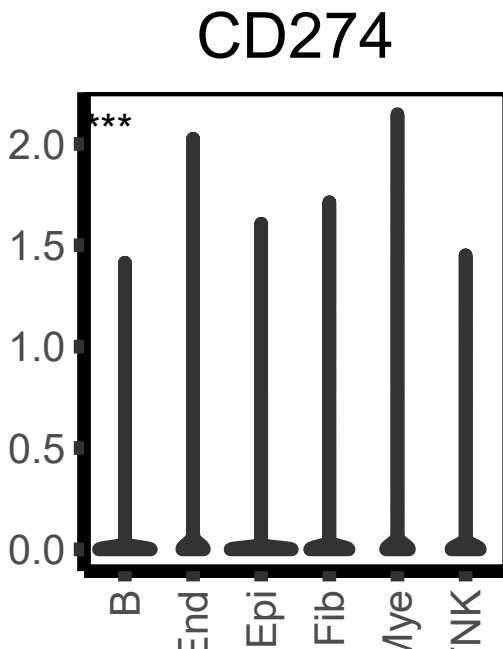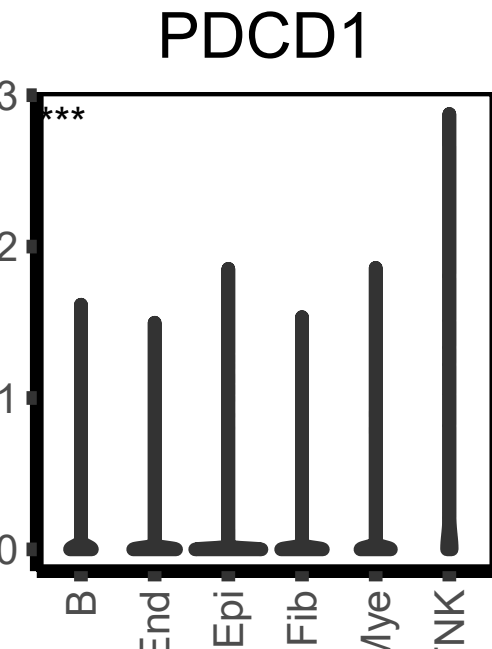

Supplement: Supplementary file 1 [file biomedicines-11-03057-s001.zip › Supplementary Figure S4.pdf]
