# Supplementary material for: Intercellular Molecular Crosstalk Networks within Invasive and Immunosuppressive Tumor Microenvironment Subtypes Associated with Clinical Outcomes in Four Cancer Types
Source: Biomedicines. 2023 Nov 14;11(11):3057. doi: 10.3390/biomedicines11113057 (PMC10669098; doi:10.3390/biomedicines11113057)
Supplement: Supplementary file 1 [file Supplementary Figure S10.pdf › Supplementary Figure S10.pdf]

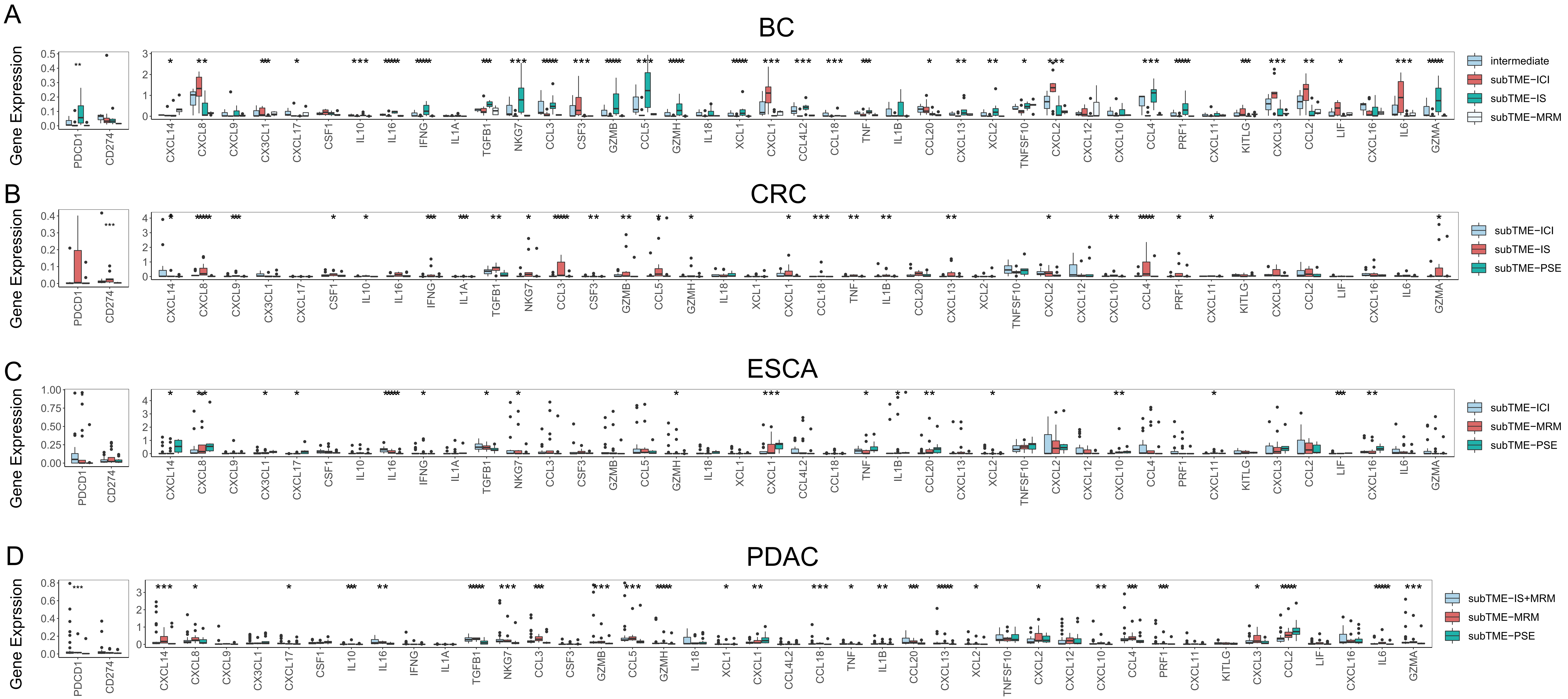

A
BC
Gene Expression
0.0 0.1 0.2 0.3 0.4 0.5
0 1 2 3
PDCD1 CD274
CXCL14 CXCL8 CXCL9 CX3CL1 CXCL17 CSF1 IL10 IL16 IFNG IL1A TGFB1 NKG7 CCL3 CSF3 GZMB CCL5 GZMH IL18 XCL1 CXCL1 CCL4L2 CCL18 TNF IL1B CCL20 CXCL13 XCL2 TNFSF10 CXCL2 CXCL12 CXCL10 CCL4 PRF1 CXCL11 KITLG CXCL3 CCL2 LIF CXCL16 IL6 GZMA
intermediate
subTME−ICI
subTME−IS
subTME−MRM
B
CRC
Gene Expression
0.0 0.1 0.2 0.3 0.4
0 1 2 3 4
PDCD1 CD274
CXCL14 CXCL8 CXCL9 CX3CL1 CXCL17 CSF1 IL10 IL16 IFNG IL1A TGFB1 NKG7 CCL3 CSF3 GZMB CCL5 GZMH IL18 XCL1 CXCL1 CCL18 TNF IL1B CCL20 CXCL13 XCL2 TNFSF10 CXCL2 CXCL12 CXCL10 CCL4 PRF1 CXCL11 KITLG CXCL3 CCL2 LIF CXCL16 IL6 GZMA
subTME−ICI
subTME−IS
subTME−PSE
C
ESCA
Gene Expression
0.00 0.25 0.50 0.75 1.00
0 1 2 3 4
PDCD1 CD274
CXCL14 CXCL8 CXCL9 CX3CL1 CXCL17 CSF1 IL10 IL16 IFNG IL1A TGFB1 NKG7 CCL3 CSF3 GZMB CCL5 GZMH IL18 XCL1 CXCL1 CCL4L2 CCL18 TNF IL1B CCL20 CXCL13 XCL2 TNFSF10 CXCL2 CXCL12 CXCL10 CCL4 PRF1 CXCL11 KITLG CXCL3 CCL2 LIF CXCL16 IL6 GZMA
subTME−ICI
subTME−MRM
subTME−PSE
D
PDAC
Gene Exprssion
0.0 0.2 0.4 0.6 0.8
0 1 2 3
PDCD1 CD274
CXCL14 CXCL8 CXCL9 CX3CL1 CXCL17 CSF1 IL10 IL16 IFNG IL1A TGFB1 NKG7 CCL3 CSF3 GZMB CCL5 GZMH IL18 XCL1 CXCL1 CCL4L2 CCL18 TNF IL1B CCL20 CXCL13 XCL2 TNFSF10 CXCL2 CXCL12 CXCL10 CCL4 PRF1 CXCL11 KITLG CXCL3 CCL2 LIF CXCL16 IL6 GZMA
subTME−IS+MRM
subTME−MRM
subTME−PSE
