# Supplementary material for: Intercellular Molecular Crosstalk Networks within Invasive and Immunosuppressive Tumor Microenvironment Subtypes Associated with Clinical Outcomes in Four Cancer Types
Source: Biomedicines. 2023 Nov 14;11(11):3057. doi: 10.3390/biomedicines11113057 (PMC10669098; doi:10.3390/biomedicines11113057)
Supplement: Supplementary file 1 [file Supplementary Figure S13.pdf › Supplementary Figure S13.pdf]

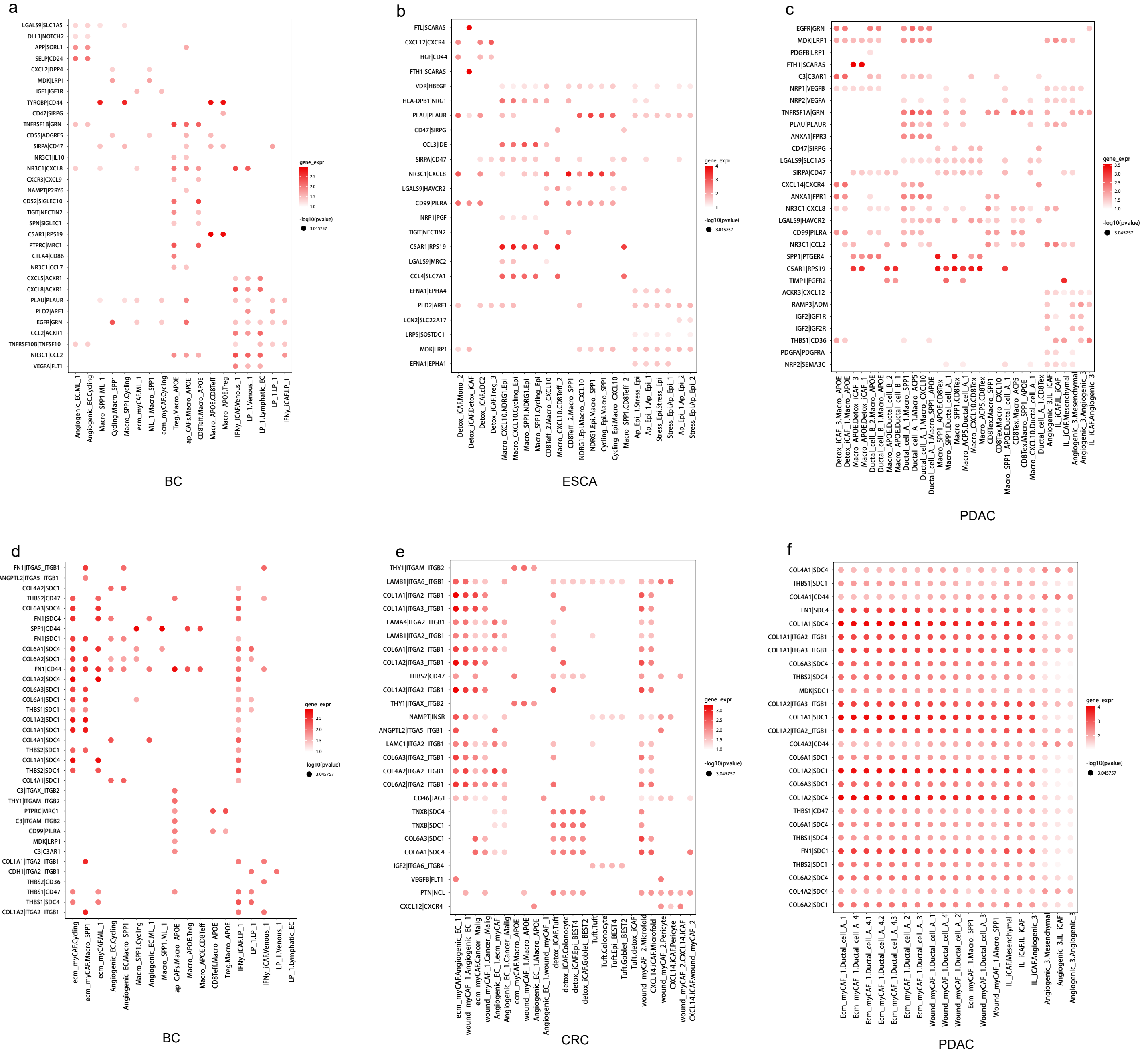
a
LGALS9|SLC1A5
DLL1|NOTCH2
APP|SORL1
SELP|CD24
CXCL2|DPP4
MDK|LRP1
IGF1|IGF1R
TYROBP|CD44
CD47|SIRPG
TNFRSF1B|GRN
CD55|ADGRE5
SIRPA|CD47
NR3C1|IL10
NR3C1|CXCL8
CXCR3|CXCL9
NAMPT|P2RY6
CD52|SIGLEC10
TIGIT|NECTIN2
SPN|SIGLEC1
C5AR1|RPS19
PTPRC|MRC1
CTLA4|CD86
NR3C1|CCL7
CXCL5|ACKR1
CXCL8|ACKR1
PLAU|PLAUR
PLD2|ARF1
EGFR|GRN
CCL2|ACKR1
TNFRSF10B|TNFSF10
NR3C1|CCL2
VEGFA|FLT1
Angiogenic_EC.ML_1
Angiogenic_EC.Cycling
Macro_SPP1.ML_1
Cycling.Macro_SPP1
Macro_SPP1.Cycling
ecm_myCAF.ML_1
ML_1.Macro_SPP1
ecm_myCAF.Cycling
Treg.Macro_APOE
ap_CAFs.Macro_APOE
CD8Teff.Macro_APOE
Macro_APOE.CD8Teff
Macro_APOE.Treg
IFNy_iCAF.Venous_1
LP_1.Venous_1
LP_1.Lymphatic_EC
LP_1.LP_1
IFNy_iCAF.LP_1
gene_expr
2.5
2.0
1.5
1.0
-log10(pvalue)
3.045757
BC
b
FTL|SCARA5
CXCL12|CXCR4
HGF|CD44
FTH1|SCARA5
VDR|HBEGF
HLA-DPB1|NRG1
PLAU|PLAUR
CD47|SIRPG
CCL3|IDE
SIRPA|CD47
NR3C1|CXCL8
LGALS9|HAVCR2
CD99|PILRA
NRP1|PGF
TIGIT|NECTIN2
C5AR1|RPS19
LGALS9|MRC2
CCL4|SLC7A1
EFNA1|EPHA4
PLD2|ARF1
LCN2|SLC22A17
LRP5|SOSTDC1
MDK|LRP1
EFNA1|EPHA1
Detox_iCAF.Mono_2
Detox_iCAF.Detox_iCAF
Detox_iCAF.cDC2
Detox_iCAF.Treg_3
Macro_CXCL10.NDRG1.Epi
Macro_CXCL10.Cycling_Epi
Macro_SPP1.NDRG1.Epi
Macro_SPP1.Cycling_Epi
CD8Teff_2.Macro_CXCL10
Macro_CXCL10.CD8Teff_2
CD8Teff_2.Macro_SPP1
NDRG1.Epi.Macro_CXCL10
NDRG1.Epi.Macro_SPP1
Cycling_Epi.Macro_SPP1
Cycling_Epi.Macro_CXCL10
Macro_SPP1.CD8Teff_2
Ap_Epi_1.Stress_Epi
Ap_Epi_1.Ap_Epi_1
Stress_Epi.Stress_Epi
Stress_Epi.Ap_Epi_1
Ap_Epi_1.Ap_Epi_2
Stress_Epi.Ap_Epi_2
gene_expr
4
3
2
1
-log10(pvalue)
3.045757
ESCA
c
EGFR|GRN
MDK|LRP1
PDGFB|LRP1
FTH1|SCARA5
C3|C3AR1
NRP1|VEGFB
NRP2|VEGFA
TNFRSF1A|GRN
PLAU|PLAUR
ANXA1|FPR3
CD47|SIRPG
LGALS9|SLC1A5
SIRPA|CD47
CXCL14|CXCR4
ANXA1|FPR1
NR3C1|CXCL8
LGALS9|HAVCR2
CD99|PILRA
NR3C1|CCL2
SPP1|PTGER4
C5AR1|RPS19
TIMP1|FGFR2
ACKR3|CXCL12
RAMP3|ADM
IGF2|IGF1R
IGF2|IGF2R
THBS1|CD36
PDGFA|PDGFRA
NRP2|SEMA3C
Detox_iCAF_3.Macro_APOE
Detox_iCAF_1.Macro_APOE
Macro_APOE.Detox_iCAF_3
Macro_APOE.Detox_iCAF_1
Ductal_cell_B_2.Macro_APOE
Ductal_cell_B_1.Macro_APOE
Macro_APOE.Ductal_cell_B_2
Macro_APOE.Ductal_cell_B_1
Ductal_cell_A_1.Macro_SPP1
Ductal_cell_A_1.Macro_ACP5
Ductal_cell_A_1.Macro_CXCL10
Ductal_cell_A_1.Macro_SPP1_APOE
Macro_SPP1_APOE.CD8Tex
Macro_SPP1.Ductal_cell_A_1
Macro_SPP1.CD8Tex
Macro_ACP5.Ductal_cell_A_1
Macro_ACP5.CD8Tex
CD8Tex.Macro_CXCL10
Macro_SPP1_APOE.Ductal_cell_A_1
CD8Tex.Macro_ACP5
CD8Tex.Macro_SPP1_APOE
Macro_CXCL10.Ductal_cell_A_1
Ductal_cell_A_1.CD8Tex
Angiogenic_3.IL_iCAF
IL_iCAF.IL_iCAF
IL_iCAF.Mesenchymal
Angiogenic_3.Mesenchymal
Angiogenic_3.Angiogenic_3
IL_iCAF.Angiogenic_3
gene_expr
3.5
3.0
2.5
2.0
1.5
1.0
-log10(pvalue)
3.045757
PDAC
d
FN1|ITGA5_ITGB1
ANGPTL2|ITGA5_ITGB1
COL4A2|SDC1
THBS2|CD47
COL6A3|SDC4
FN1|SDC4
SPP1|CD44
FN1|SDC1
COL6A1|SDC4
COL6A2|SDC1
FN1|CD44
COL1A2|SDC4
COL6A3|SDC1
COL6A1|SDC1
THBS1|SDC1
COL1A2|SDC1
COL1A1|SDC1
COL4A1|SDC4
THBS2|SDC1
COL1A1|SDC4
THBS2|SDC4
COL4A1|SDC1
C3|ITGAX_ITGB2
THY1|ITGAM_ITGB2
PTPRC|MRC1
C3|ITGAM_ITGB2
CD99|PILRA
MDK|LRP1
C3|C3AR1
COL1A1|ITGA2_ITGB1
CDH1|ITGA2_ITGB1
THBS2|CD36
THBS1|CD47
THBS1|SDC4
COL1A2|ITGA2_ITGB1
ecm_myCAF.Cycling
ecm_myCAF.Macro_SPP1
ecm_myCAF.ML_1
Angiogenic_EC.Cycling
Angiogenic_EC.Macro_SPP1
Macro_SPP1.Cycling
Angiogenic_EC.ML_1
Macro_SPP1.ML_1
ap_CAFs.Macro_APOE
Macro_APOE.Treg
Macro_APOE.CD8Teff
CD8Teff.Macro_APOE
Treg.Macro_APOE
IFNy_iCAF.LP_1
LP_1.LP_1
IFNy_iCAF.Venous_1
LP_1.Venous_1
LP_1.Lymphatic_EC
gene_expr
2.5
2.0
1.5
1.0
-log10(pvalue)
3.045757
BC
e
THY1|ITGAM_ITGB2
LAMB1|ITGA6_ITGB1
COL1A1|ITGA2_ITGB1
COL1A1|ITGA3_ITGB1
LAMA4|ITGA2_ITGB1
LAMB1|ITGA2_ITGB1
COL6A1|ITGA2_ITGB1
COL1A2|ITGA3_ITGB1
THBS2|CD47
COL1A2|ITGA2_ITGB1
THY1|ITGAX_ITGB2
NAMPT|INSR
ANGPTL2|ITGA5_ITGB1
LAMC1|ITGA2_ITGB1
COL6A3|ITGA2_ITGB1
COL4A2|ITGA2_ITGB1
COL6A2|ITGA2_ITGB1
CD46|JAG1
TNXB|SDC4
TNXB|SDC1
COL6A3|SDC1
COL6A1|SDC4
IGF2|ITGA6_ITGB4
VEGFB|FLT1
PTN|NCL
CXCL12|CXCR4
ecm_myCAF.Angiogenic_EC_1
wound_myCAF_1.Angiogenic_EC_1
ecm_myCAF.Cancer_Malig
wound_myCAF_1.Cancer_Malig
Angiogenic_EC_1.ecm_myCAF
Angiogenic_EC_1.Cancer_Malig
ecm_myCAF.Macro_APOE
wound_myCAF_1.Macro_APOE
Angiogenic_EC_1.Macro_APOE
Angiogenic_EC_1.wound_myCAF_1
detox_iCAF.Tuft
detox_iCAF.Colonocyte
detox_iCAF.Epi_BEST4
detox_iCAF.Goblet_BEST2
Tuft.Tuft
Tuft.Colonocyte
Tuft.Epi_BEST4
Tuft.Goblet_BEST2
Tuft.detox_iCAF
wound_myCAF_2.Microfold
CXCL14.iCAF.Microfold
wound_myCAF_2.Pericyte
CXCL14.iCAF.Pericyte
wound_myCAF_2.CXCL14.iCAF
CXCL14.iCAF.wound_myCAF_2
gene_expr
3.0
2.5
2.0
1.5
1.0
-log10(pvalue)
3.045757
CRC
f
COL4A1|SDC4
THBS1|SDC1
COL4A1|CD44
FN1|SDC4
COL1A1|SDC4
COL1A1|ITGA2_ITGB1
COL1A1|ITGA3_ITGB1
COL6A3|SDC4
THBS2|SDC4
MDK|SDC1
COL1A2|ITGA3_ITGB1
COL1A1|SDC1
COL1A2|ITGA2_ITGB1
COL4A2|CD44
COL6A1|SDC1
COL1A2|SDC1
COL6A3|SDC1
COL1A2|SDC4
THBS1|CD47
COL6A1|SDC4
THBS1|SDC4
FN1|SDC1
THBS2|SDC1
COL6A2|SDC4
COL4A2|SDC4
COL6A2|SDC1
Ecm_myCAF_1.Ductal_cell_A_1
Ecm_myCAF_1.Ductal_cell_A_4
Ecm_myCAF_1.Ductal_cell_A_4.1
Ecm_myCAF_1.Ductal_cell_A_4.2
Ecm_myCAF_1.Ductal_cell_A_4.3
Ecm_myCAF_1.Ductal_cell_A_2
Ecm_myCAF_1.Ductal_cell_A_3
Wound_myCAF_1.Ductal_cell_A_1
Wound_myCAF_1.Ductal_cell_A_4
Wound_myCAF_1.Ductal_cell_A_2
Ecm_myCAF_1.Macro_SPP1
Wound_myCAF_1.Ductal_cell_A_3
Wound_myCAF_1.Macro_SPP1
IL_iCAF.Mesenchymal
IL_iCAF.IL_iCAF
IL_iCAF.Angiogenic_3
Angiogenic_3.Mesenchymal
Angiogenic_3.IL_iCAF
Angiogenic_3.Angiogenic_3
gene_expr
4
3
2
-log10(pvalue)
3.045757
PDAC
