# Supplementary material for: Intercellular Molecular Crosstalk Networks within Invasive and Immunosuppressive Tumor Microenvironment Subtypes Associated with Clinical Outcomes in Four Cancer Types
Source: Biomedicines. 2023 Nov 14;11(11):3057. doi: 10.3390/biomedicines11113057 (PMC10669098; doi:10.3390/biomedicines11113057)
Supplement: Supplementary file 1 [file Supplementary Figure S5.pdf › Supplementary Figure S5.pdf]

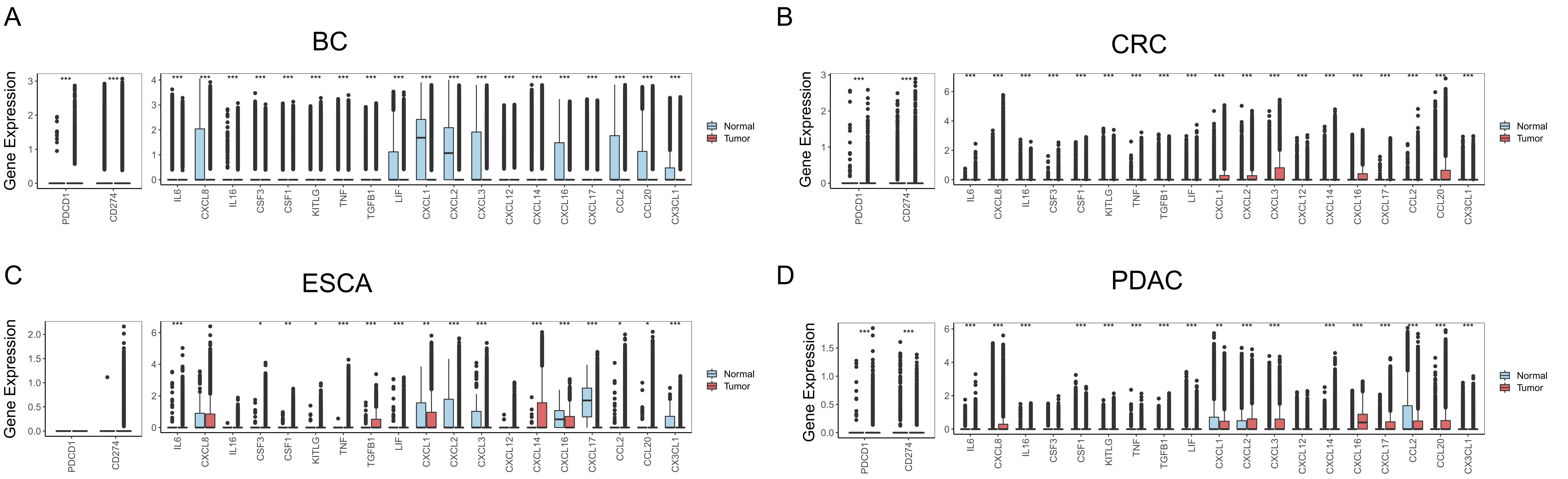
A
BC
B
CRC
C
ESCA
D
PDAC
Gene Expression
PDCD1
CD274
IL6
CXCL8
IL16
CSF3
CSF1
KITLG
TNF
TGFB1
LIF
CXCL1
CXCL2
CXCL3
CXCL12
CXCL14
CXCL16
CXCL17
CCL2
CCL20
CX3CL1
Normal
Tumor
