# Supplementary material for: Intercellular Molecular Crosstalk Networks within Invasive and Immunosuppressive Tumor Microenvironment Subtypes Associated with Clinical Outcomes in Four Cancer Types
Source: Biomedicines. 2023 Nov 14;11(11):3057. doi: 10.3390/biomedicines11113057 (PMC10669098; doi:10.3390/biomedicines11113057)
Supplement: Supplementary file 1 [file Supplementary Figure S6.pdf › Supplementary Figure S6.pdf]

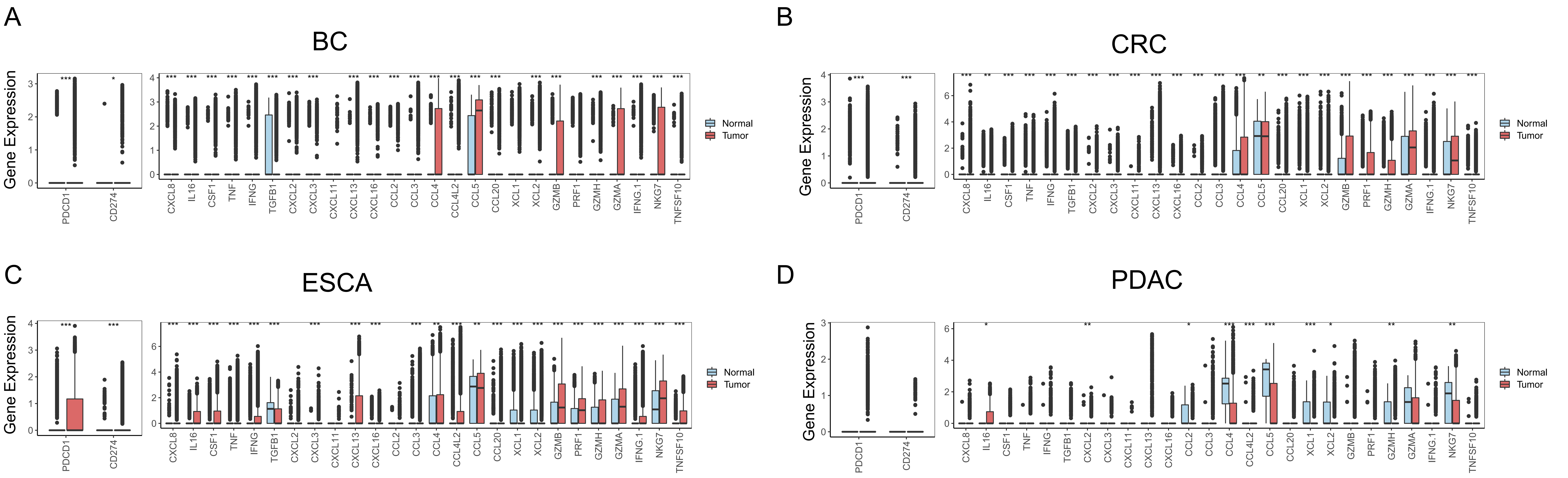
A
BC
B
CRC
C
ESCA
D
PDAC
Gene Expression
Normal
Tumor
PDCD1
CD274
CXCL8
IL16
CSF1
TNF
IFNG
TGFB1
CXCL2
CXCL3
CXCL11
CXCL13
CXCL16
CCL2
CCL3
CCL4
CCL4L2
CCL5
CCL20
XCL1
XCL2
GZMB
PRF1
GZMH
GZMA
IFNG.1
NKG7
TNFSF10
