# Supplementary material for: Intercellular Molecular Crosstalk Networks within Invasive and Immunosuppressive Tumor Microenvironment Subtypes Associated with Clinical Outcomes in Four Cancer Types
Source: Biomedicines. 2023 Nov 14;11(11):3057. doi: 10.3390/biomedicines11113057 (PMC10669098; doi:10.3390/biomedicines11113057)
Supplement: Supplementary file 1 [file Supplementary Figure S7.pdf › Supplementary Figure S7.pdf]

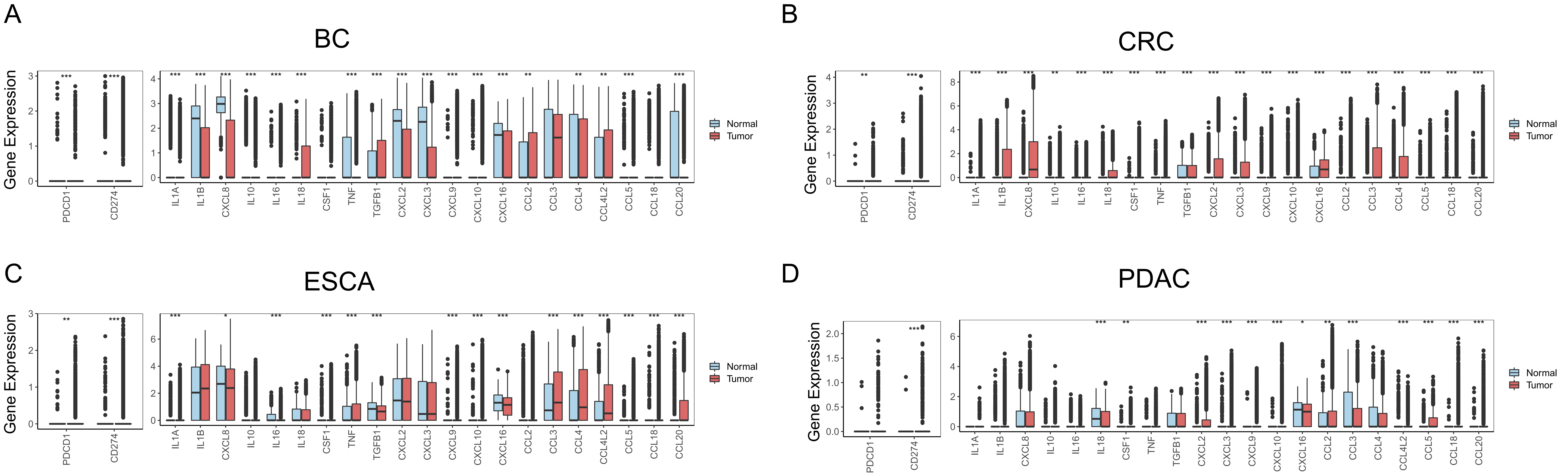
A
BC
B
CRC
C
ESCA
D
PDAC
Gene Expression
PDCD1
CD274
IL1A
IL1B
CXCL8
IL10
IL16
IL18
CSF1
TNF
TGFB1
CXCL2
CXCL3
CXCL9
CXCL10
CXCL16
CCL2
CCL3
CCL4
CCL4L2
CCL5
CCL18
CCL20
Normal
Tumor
